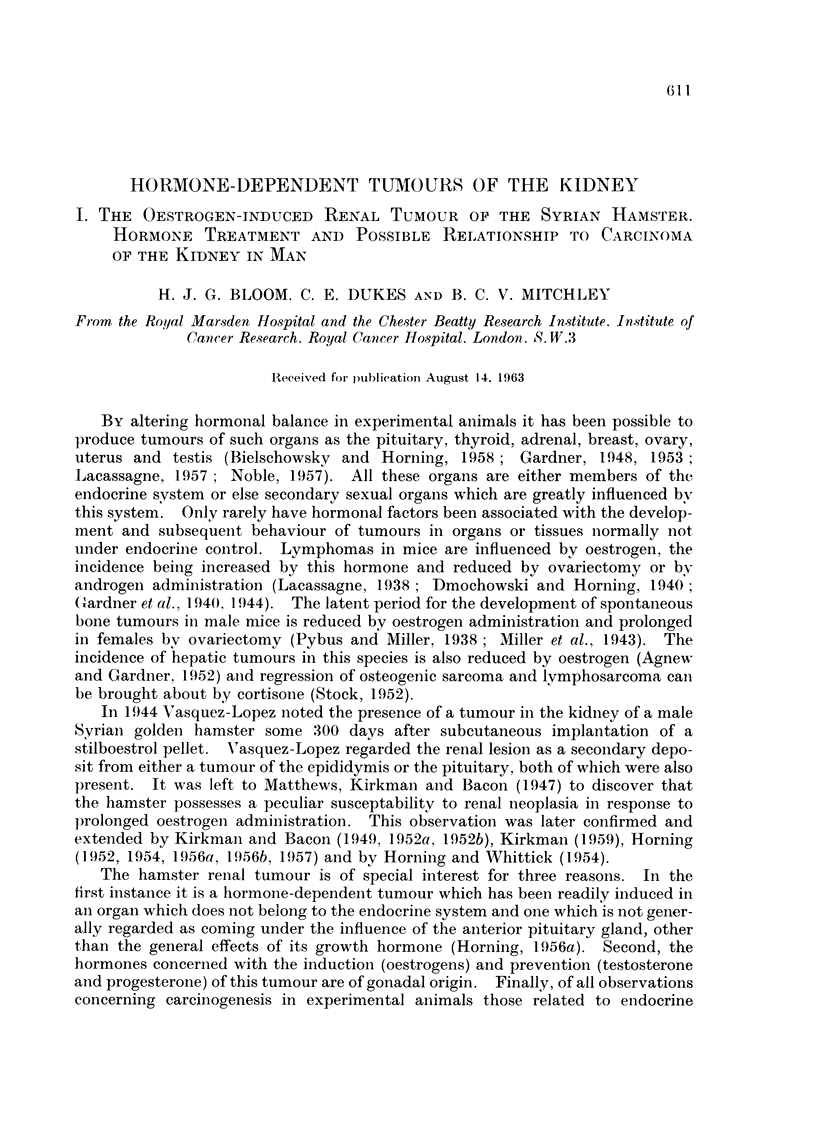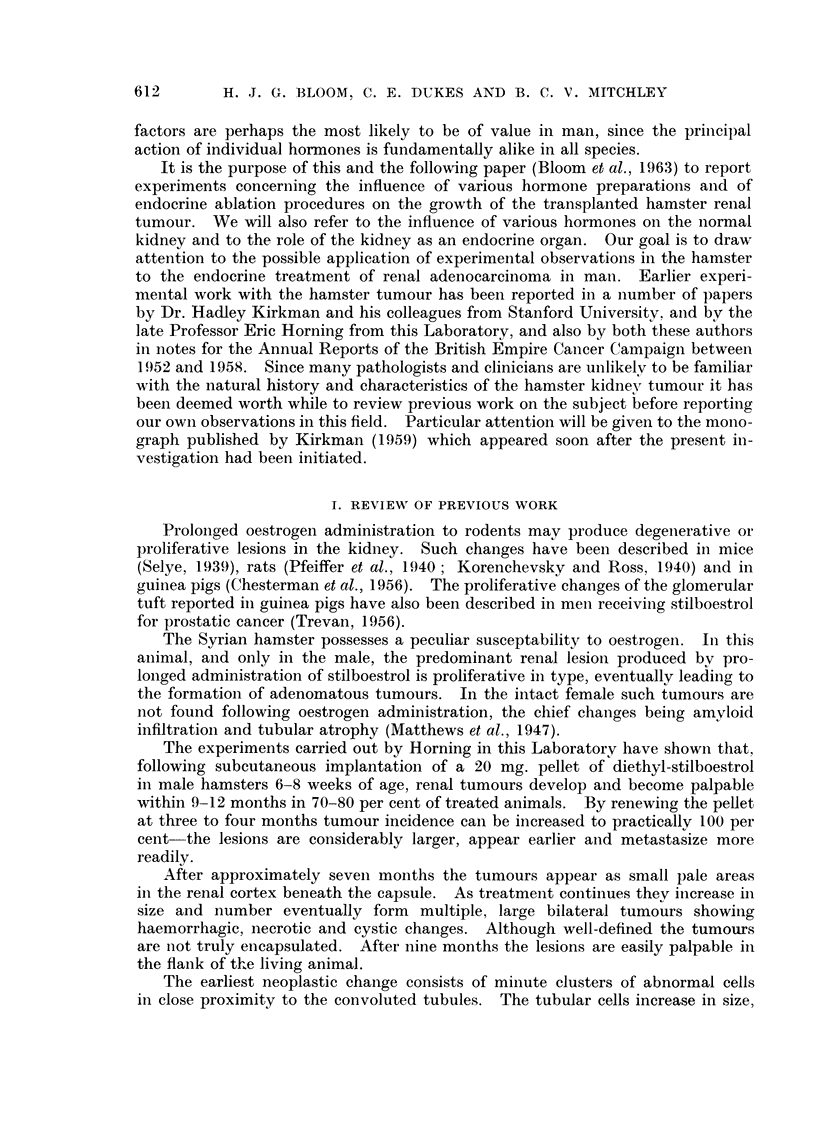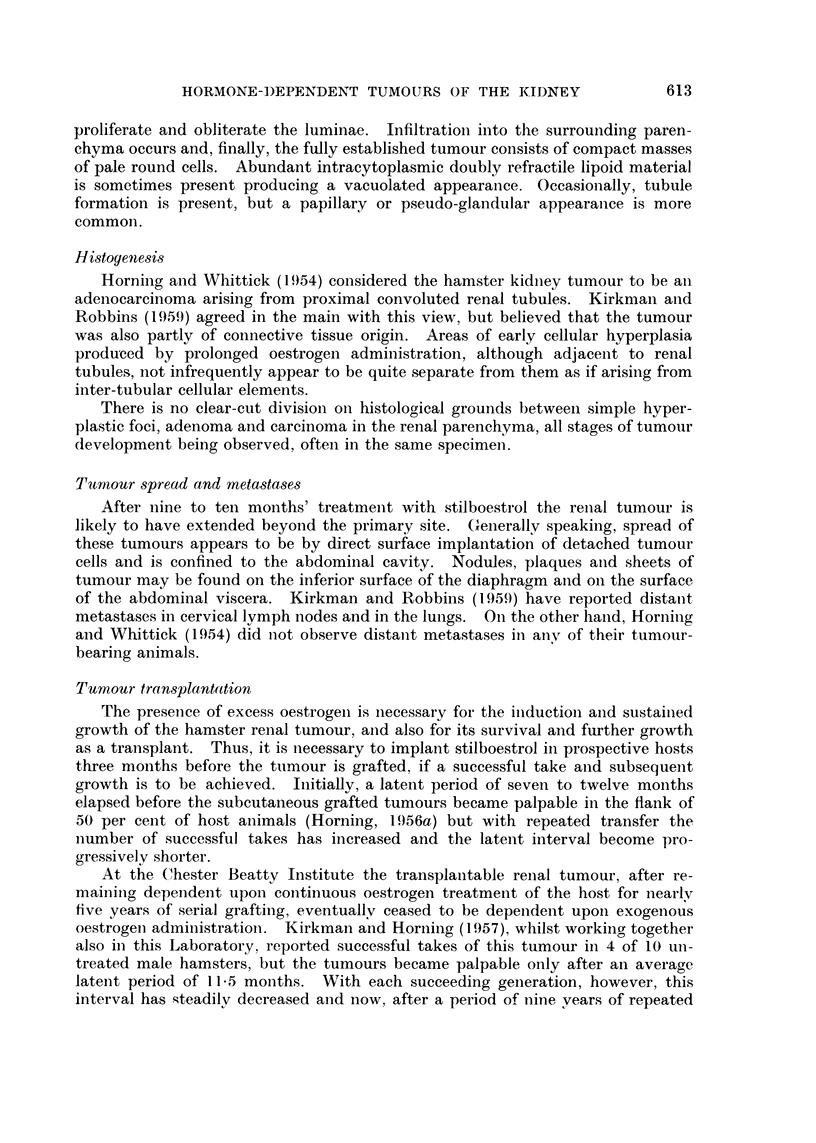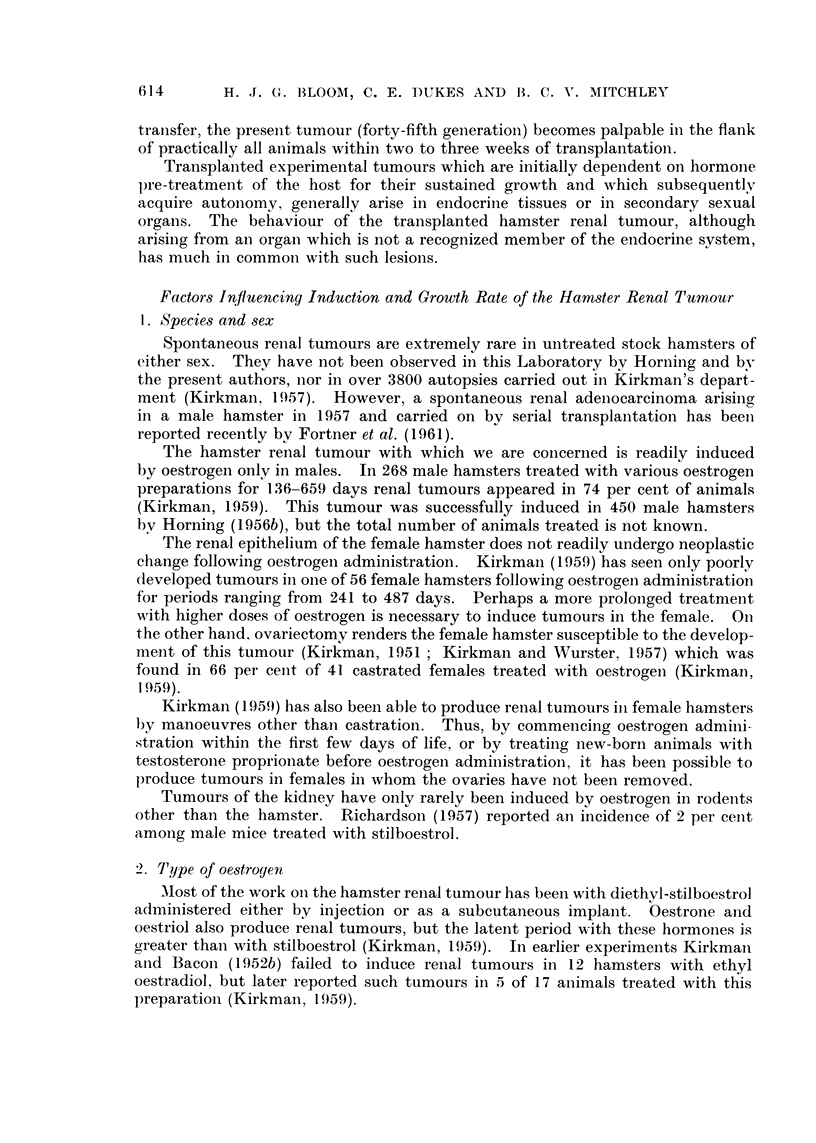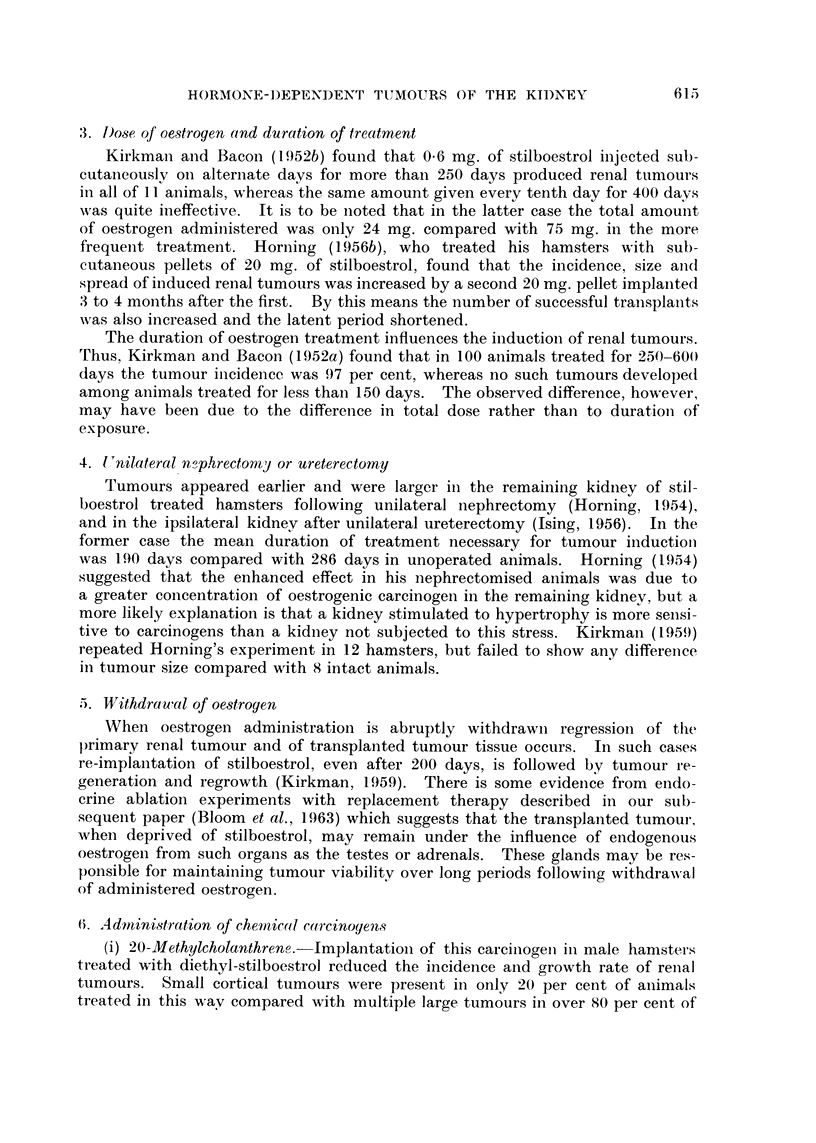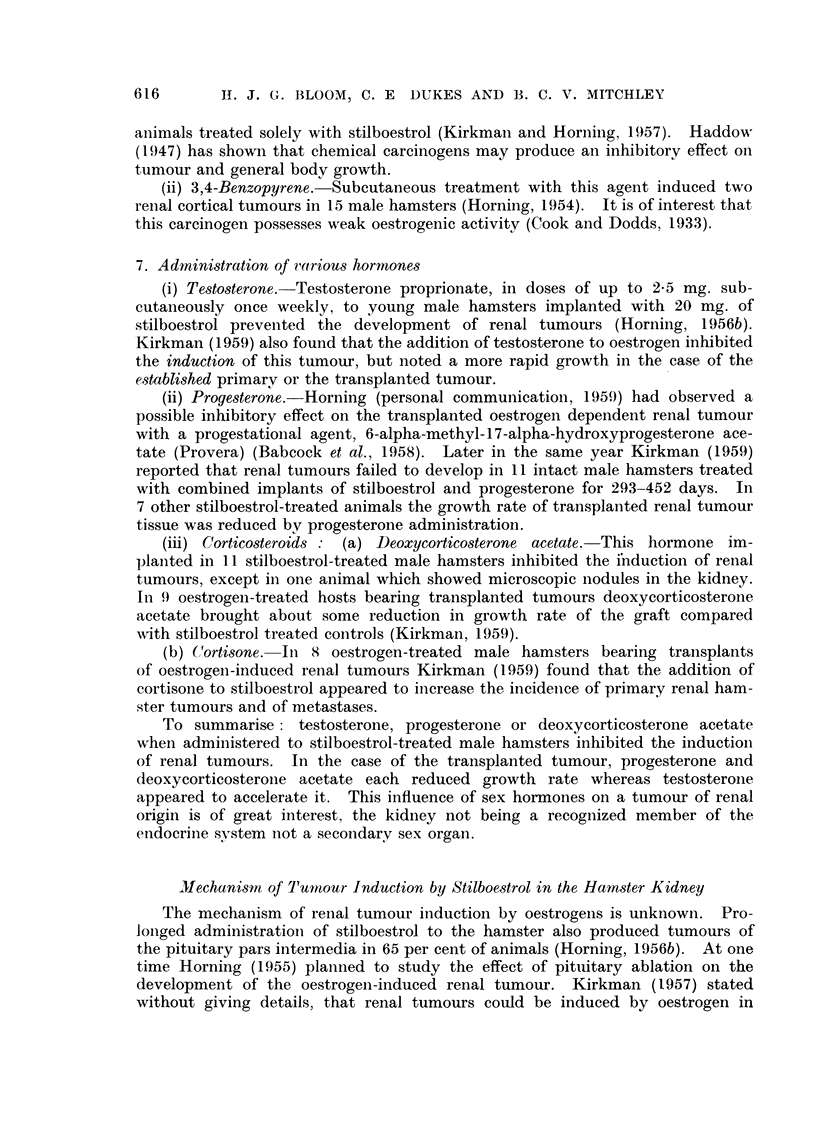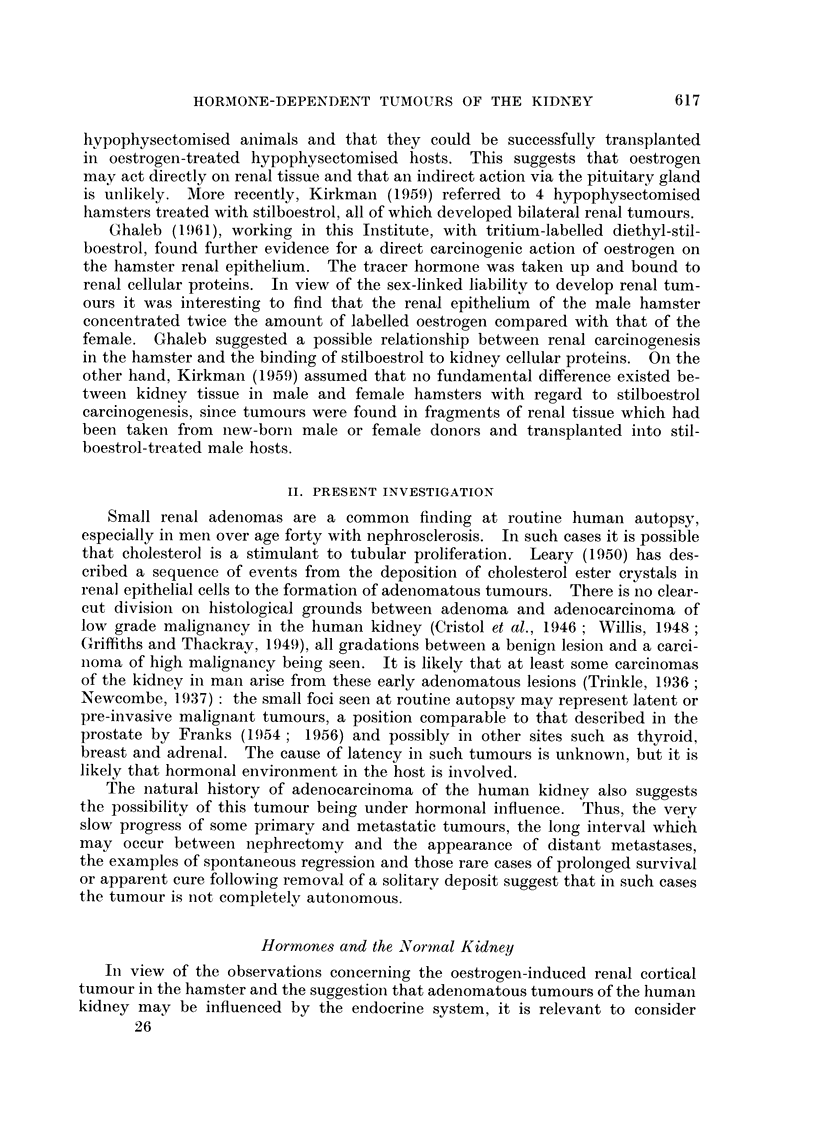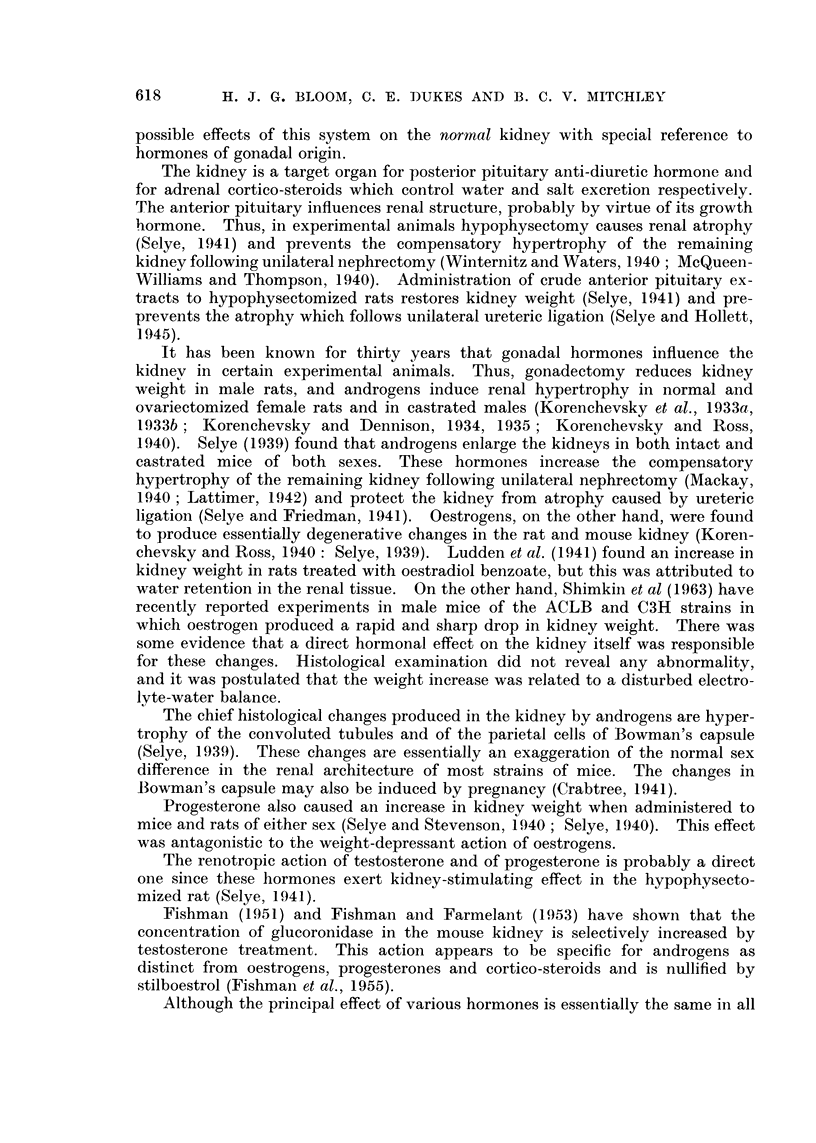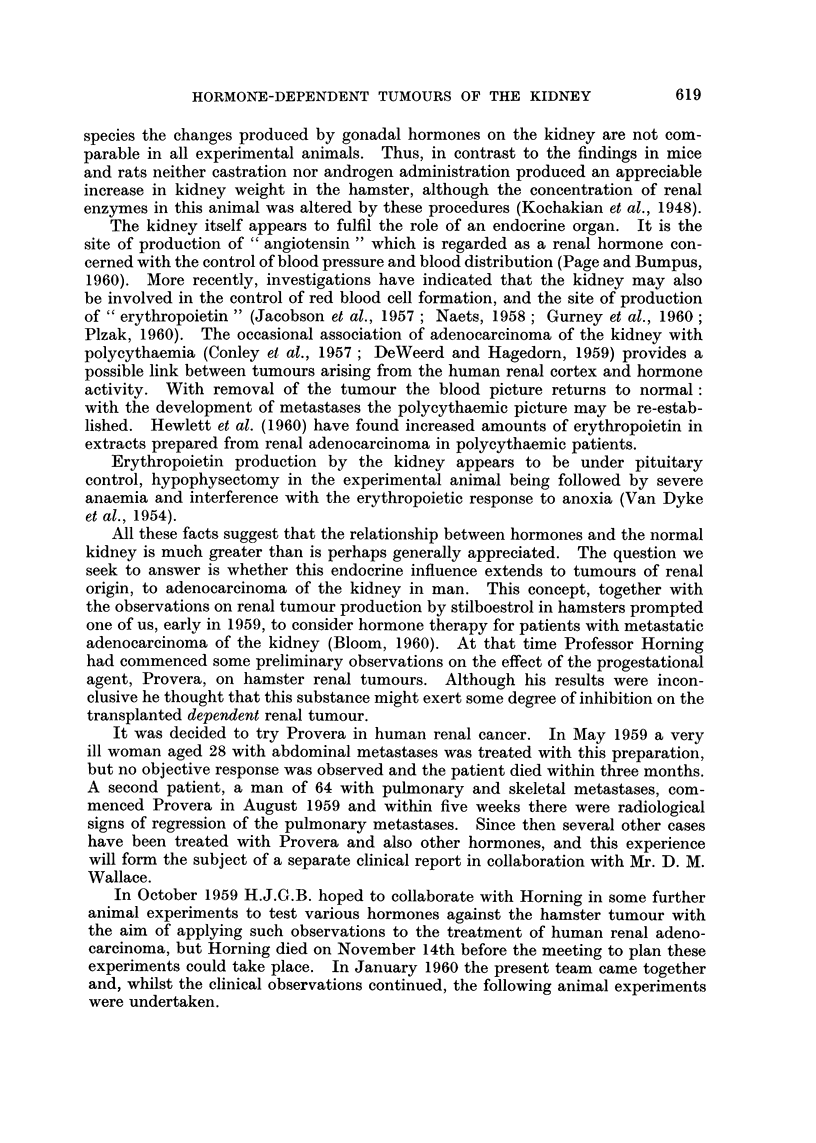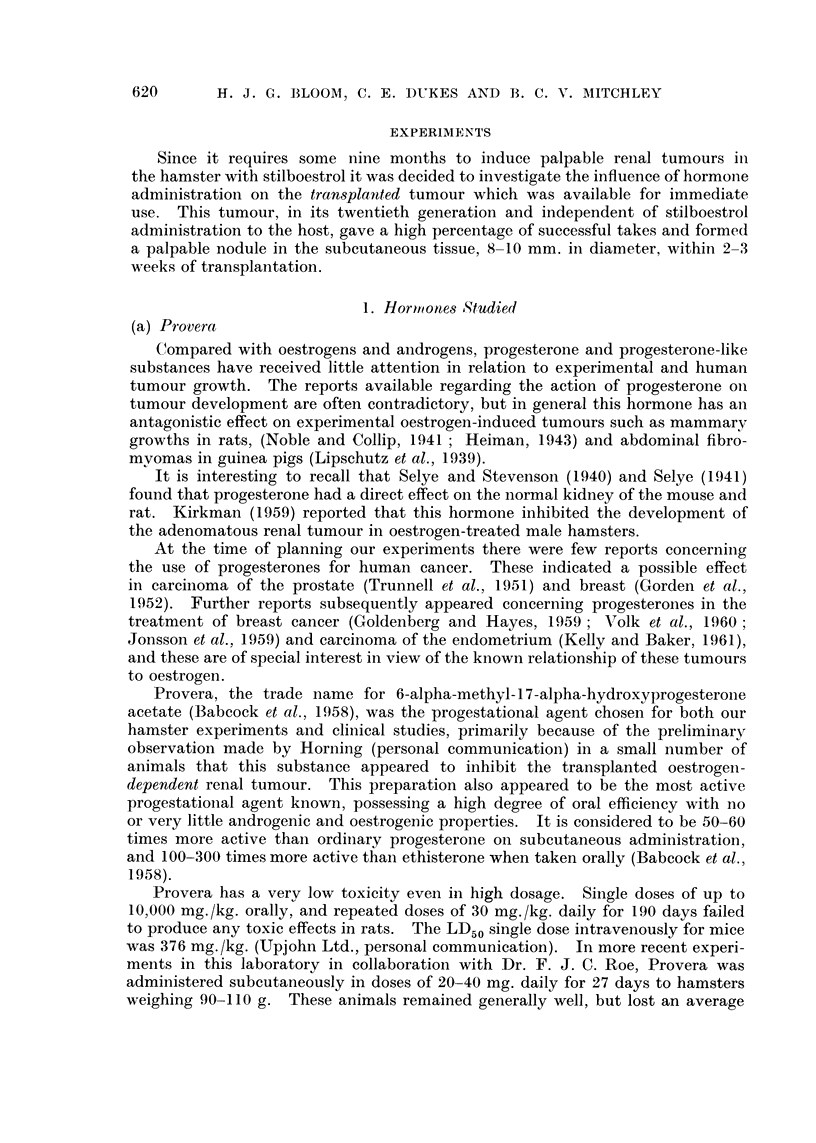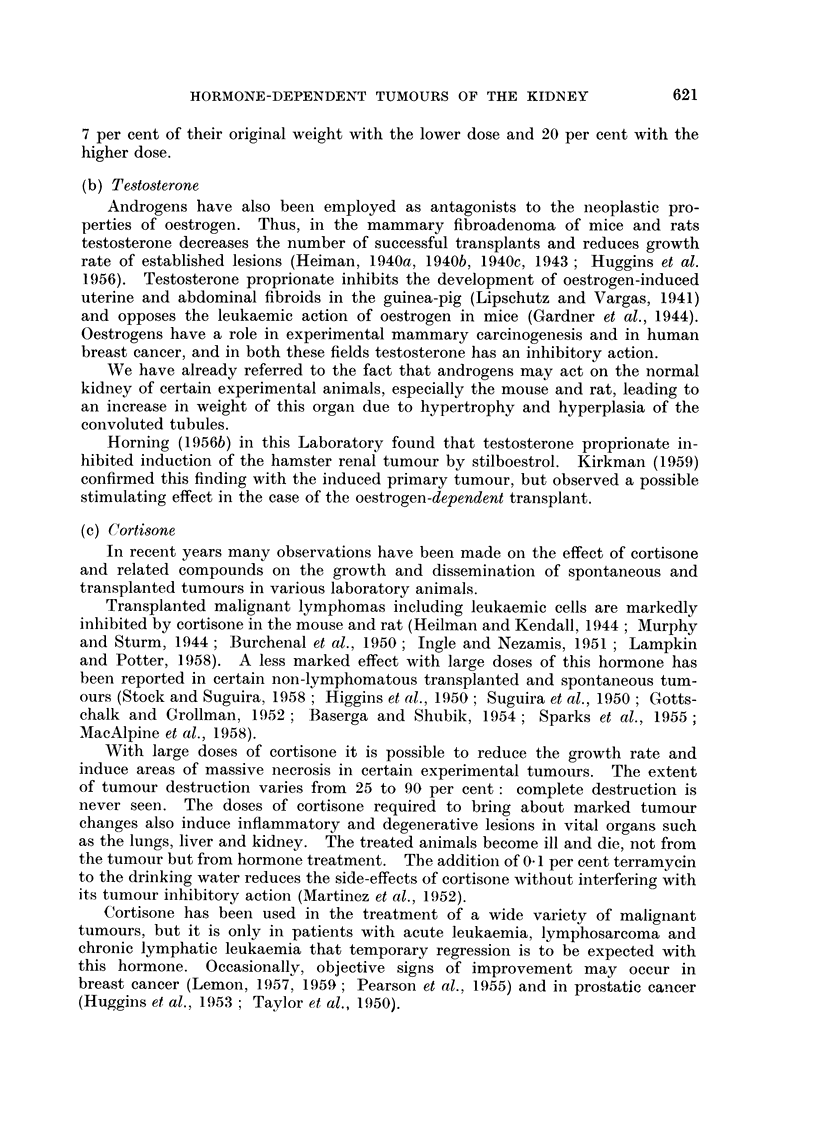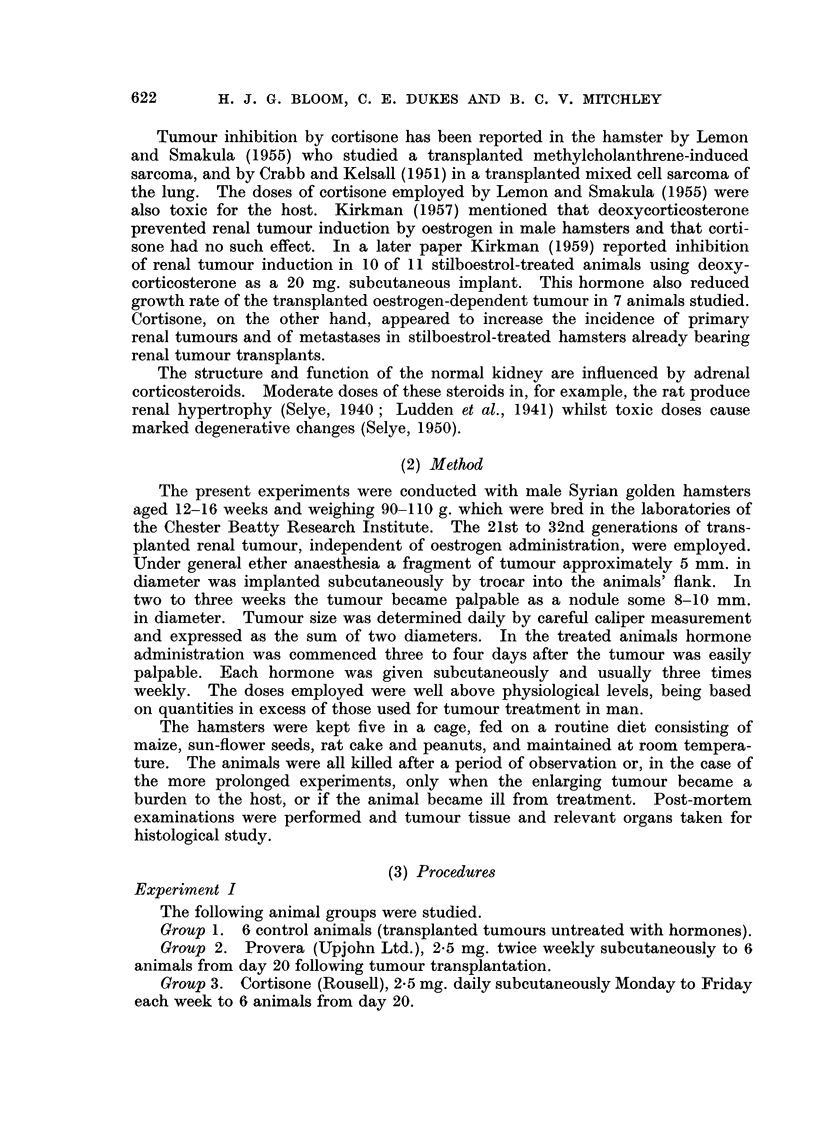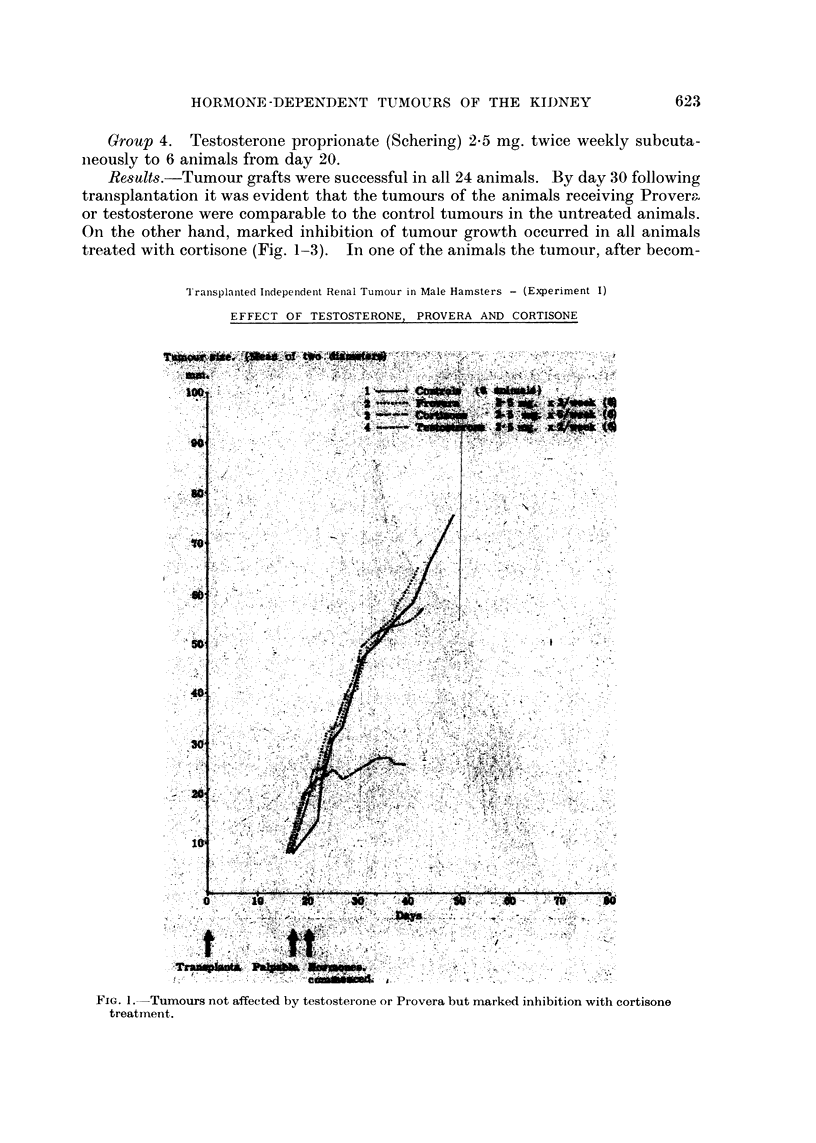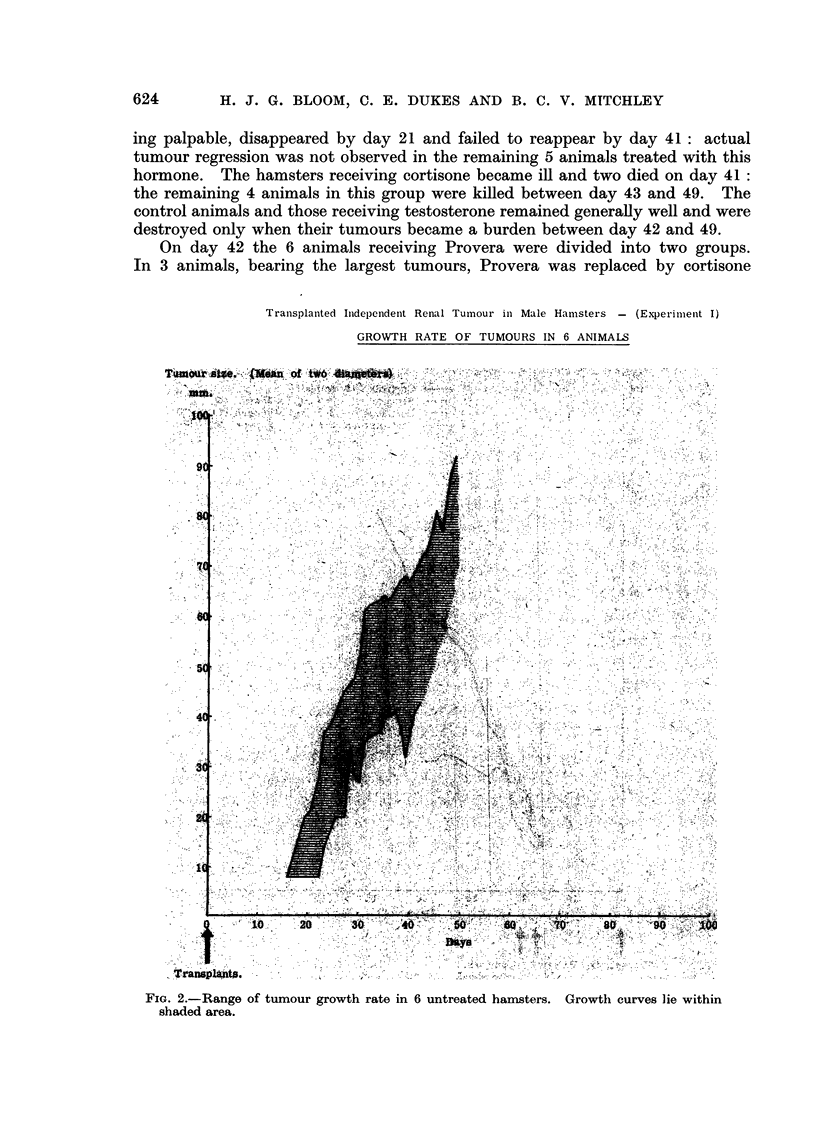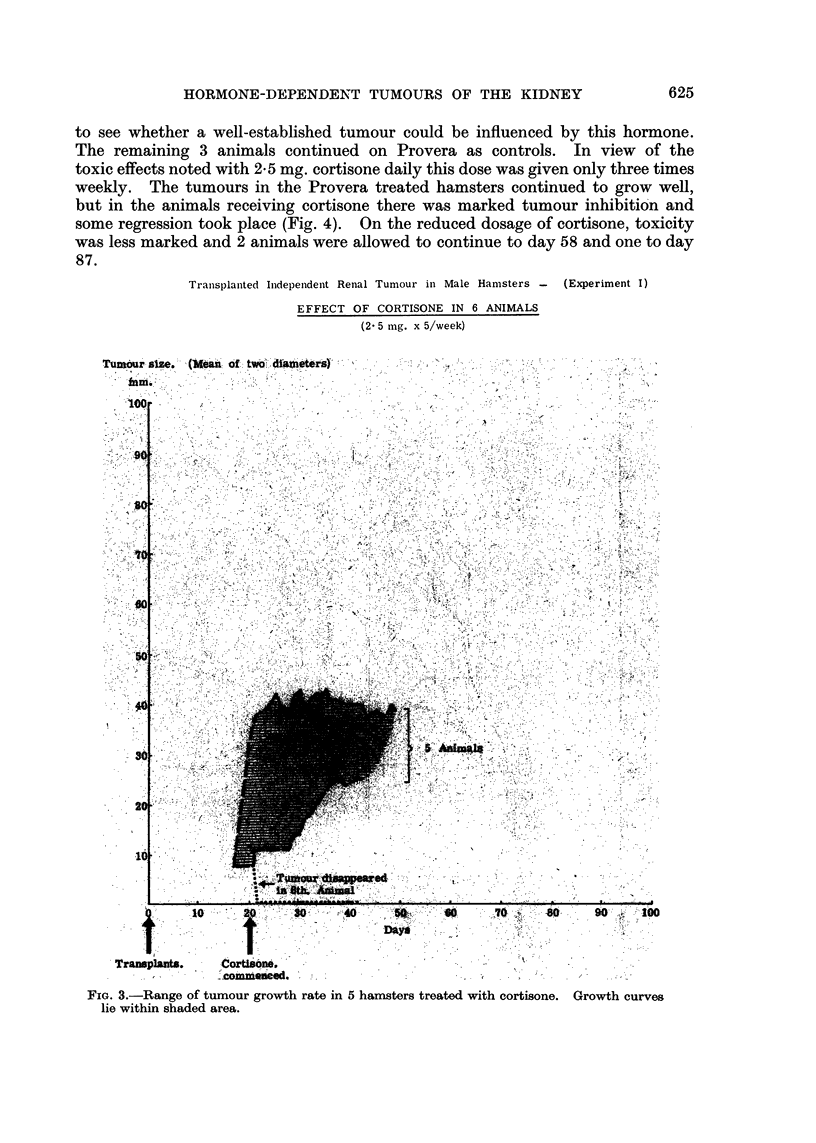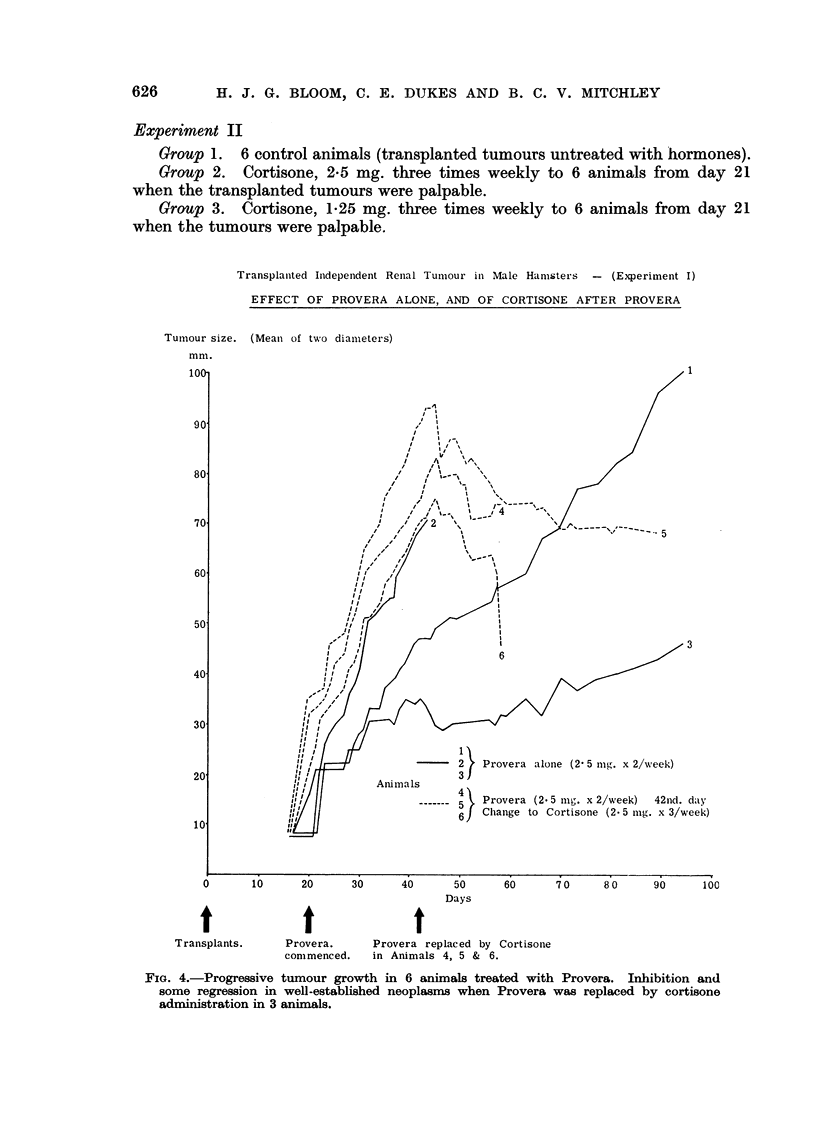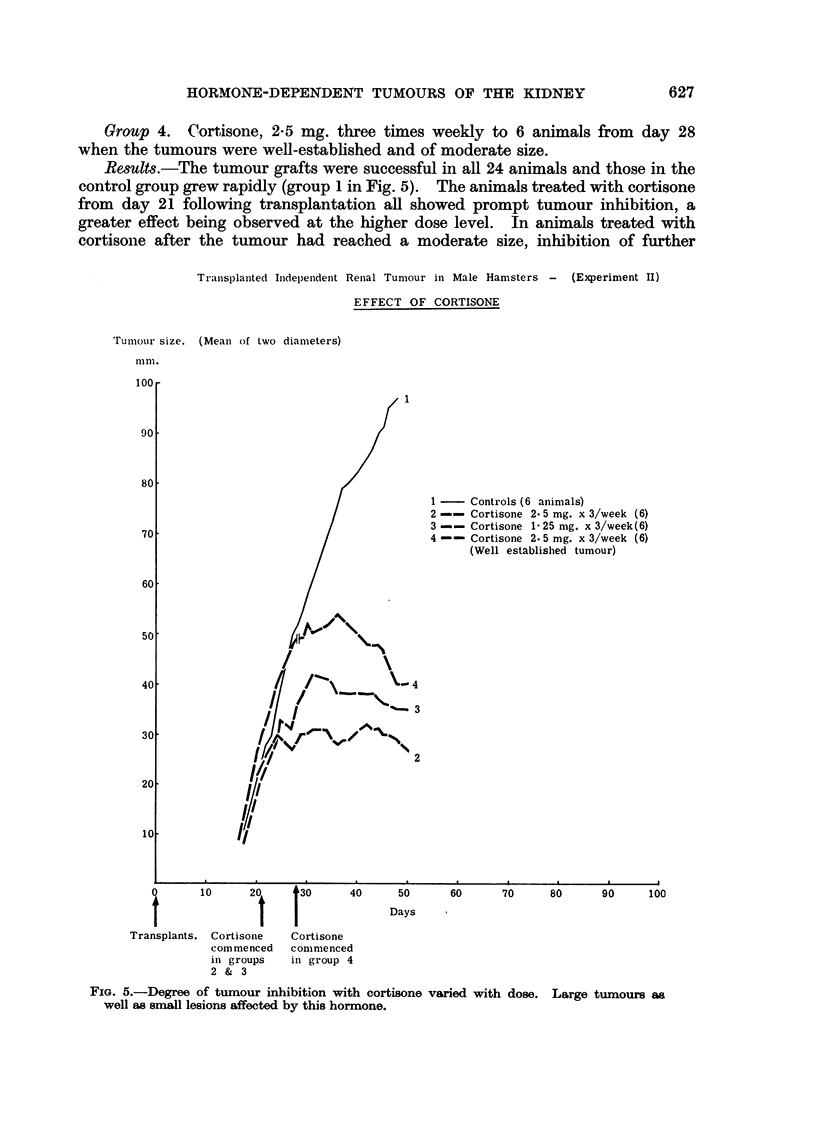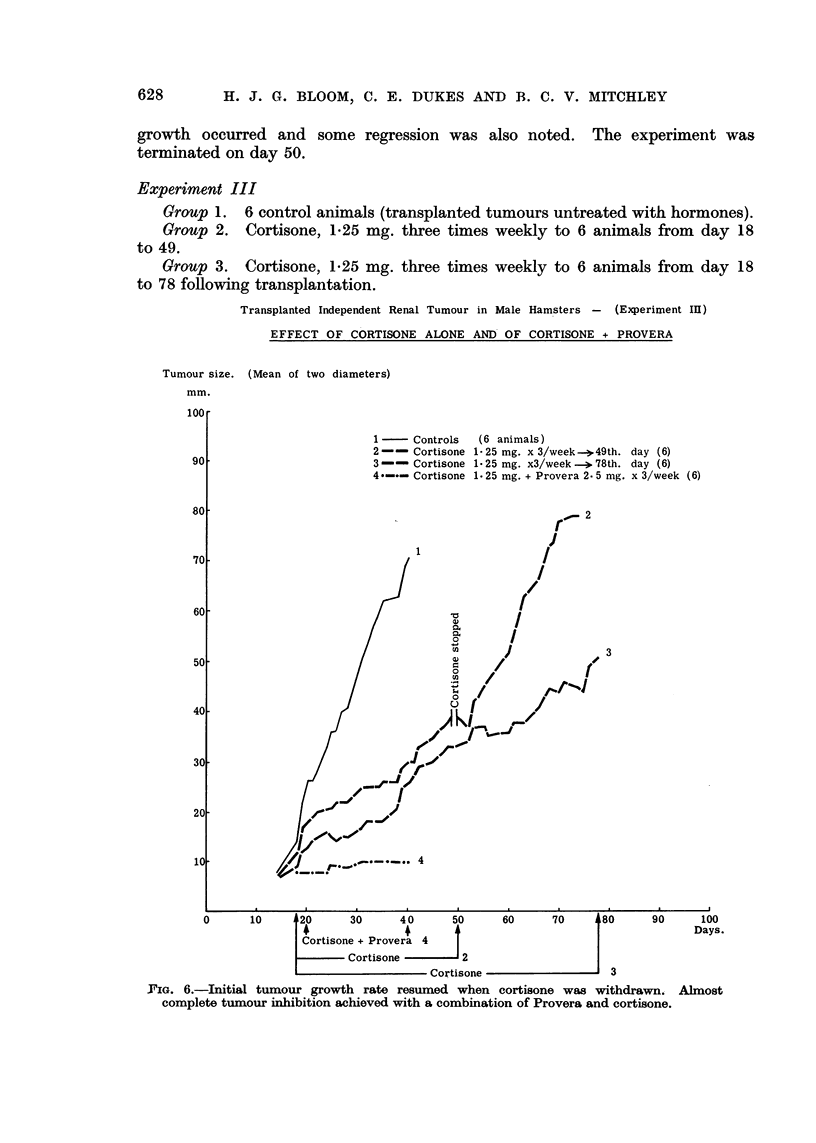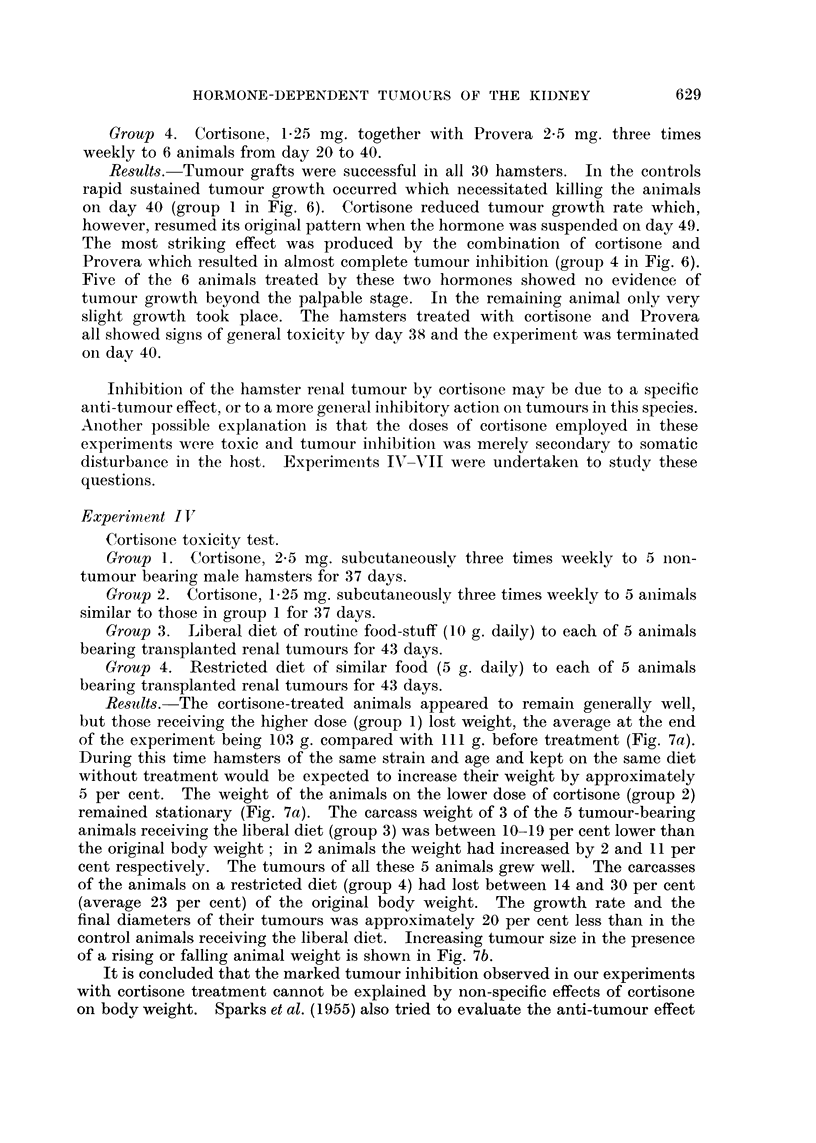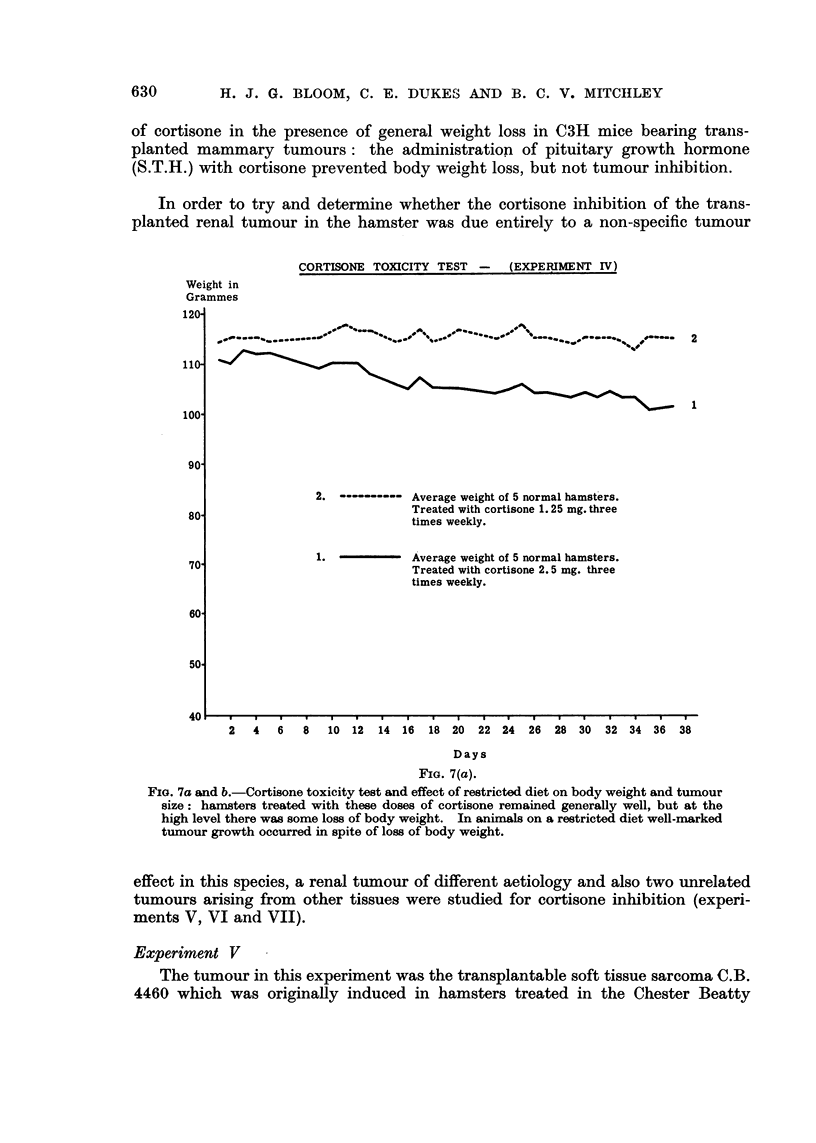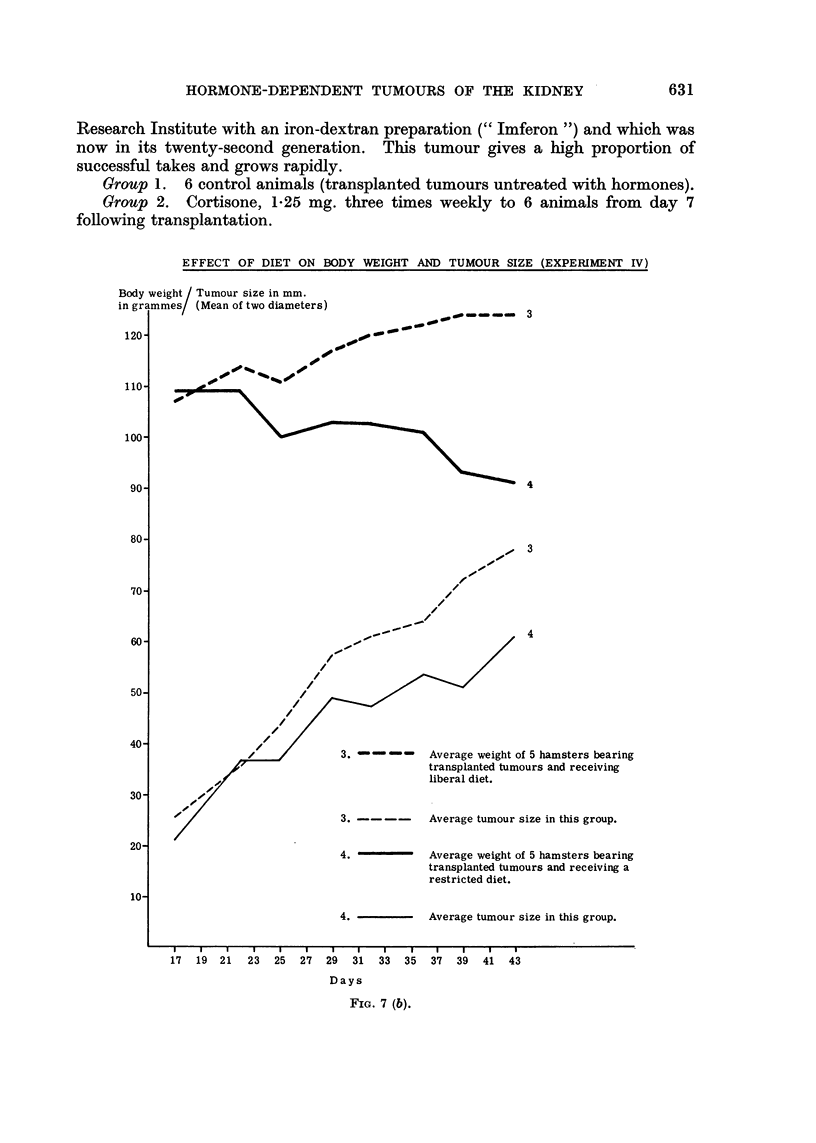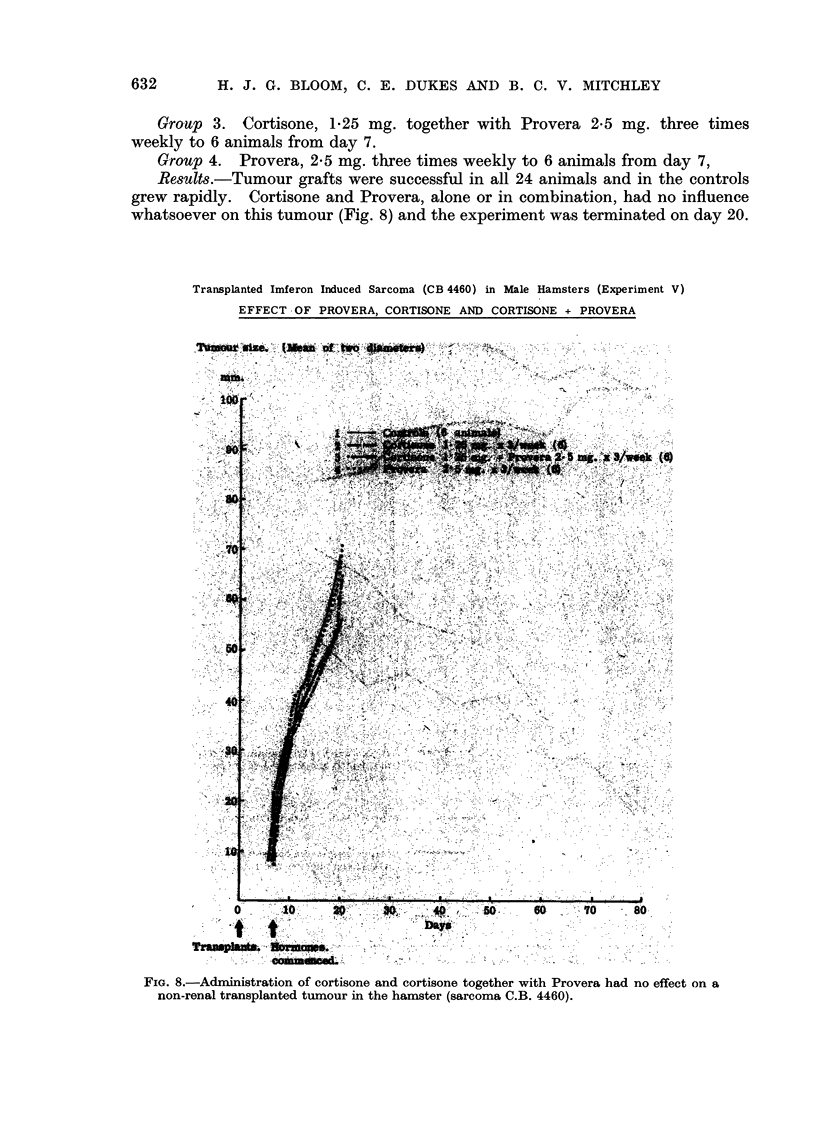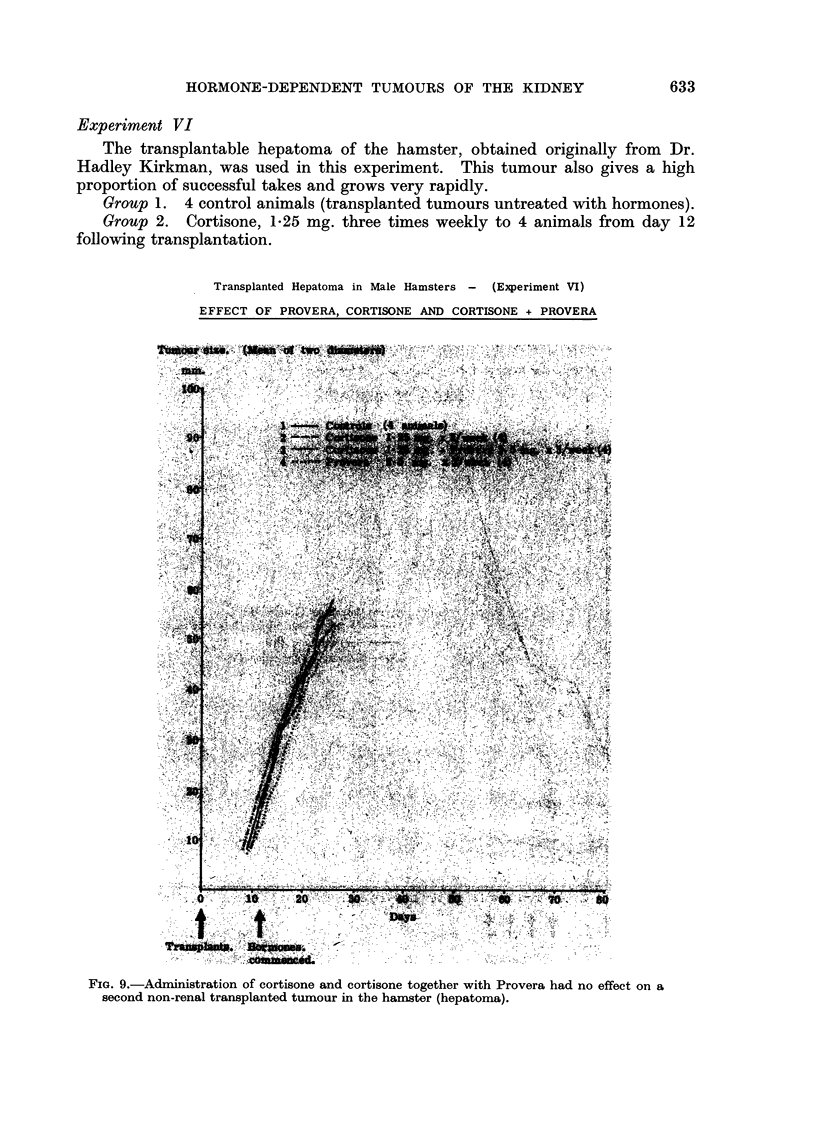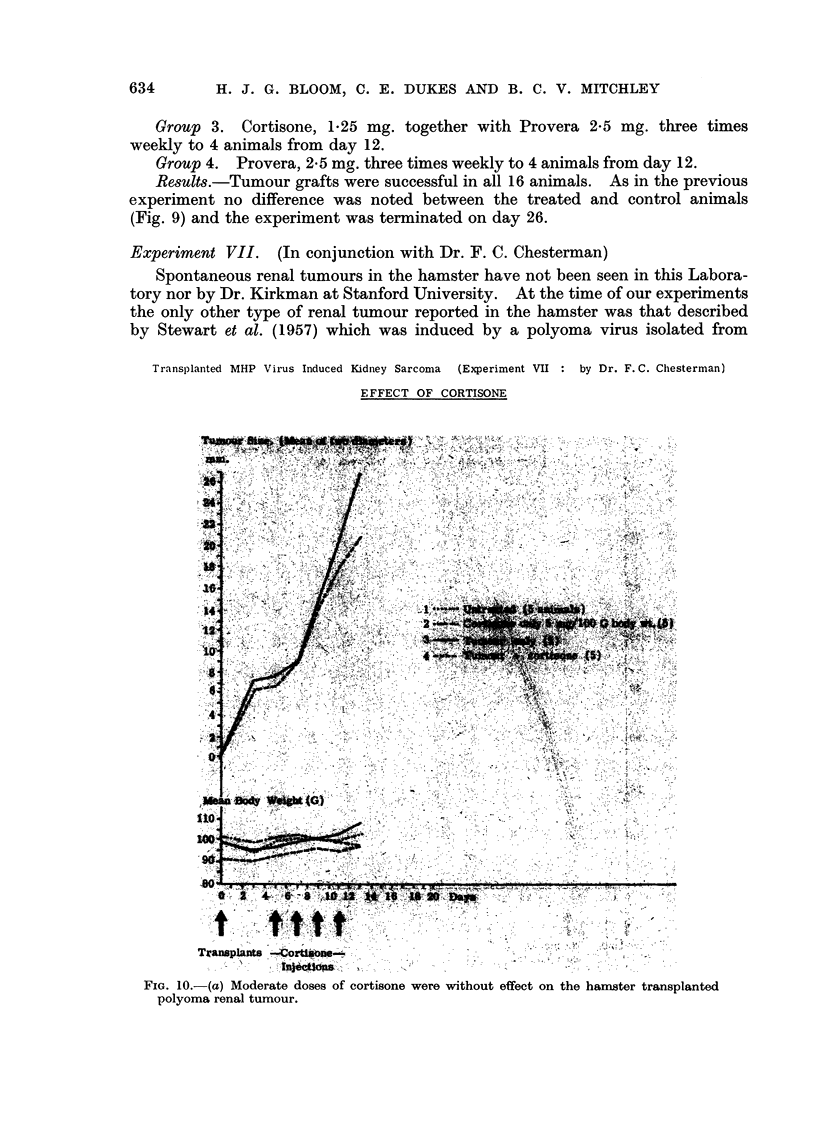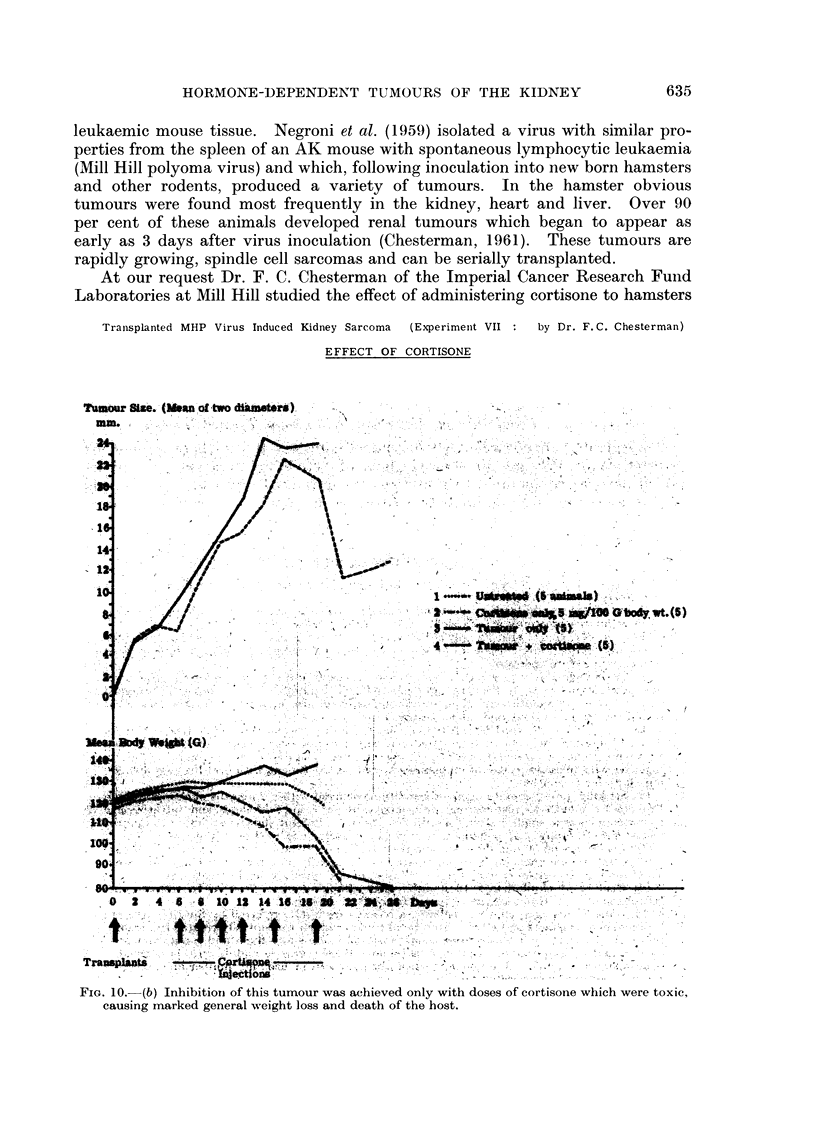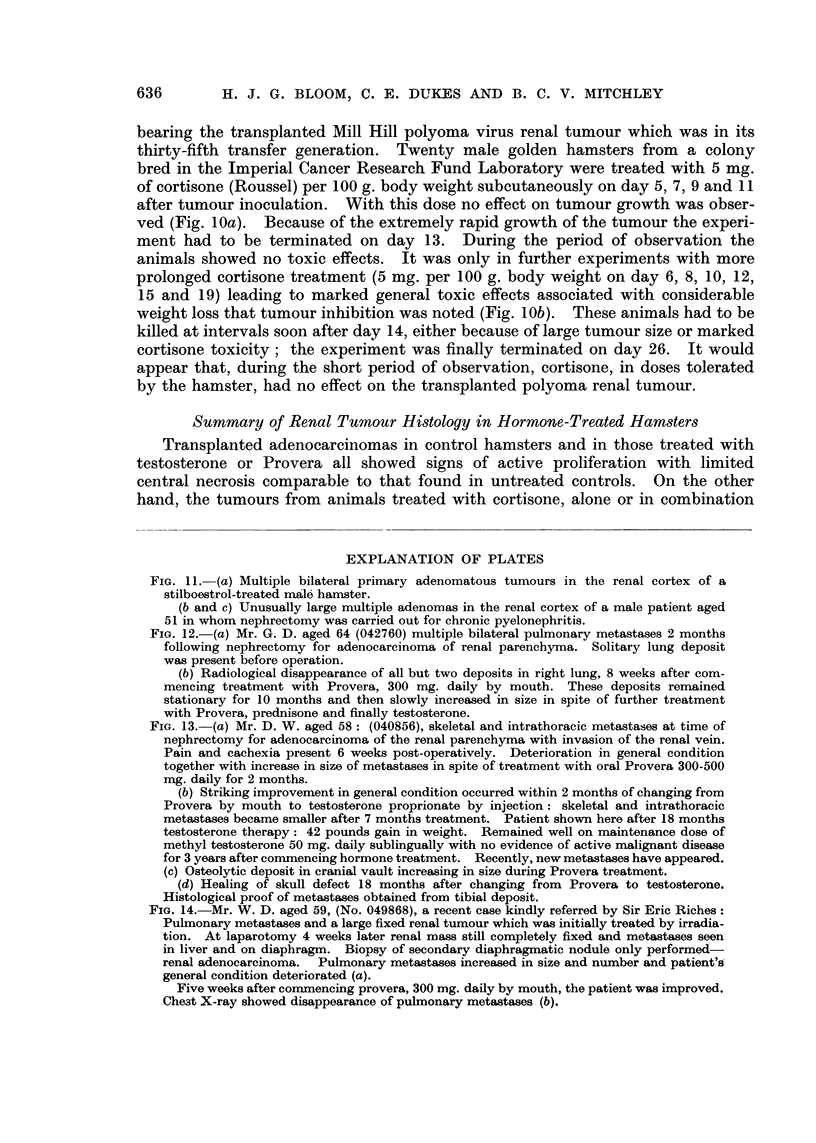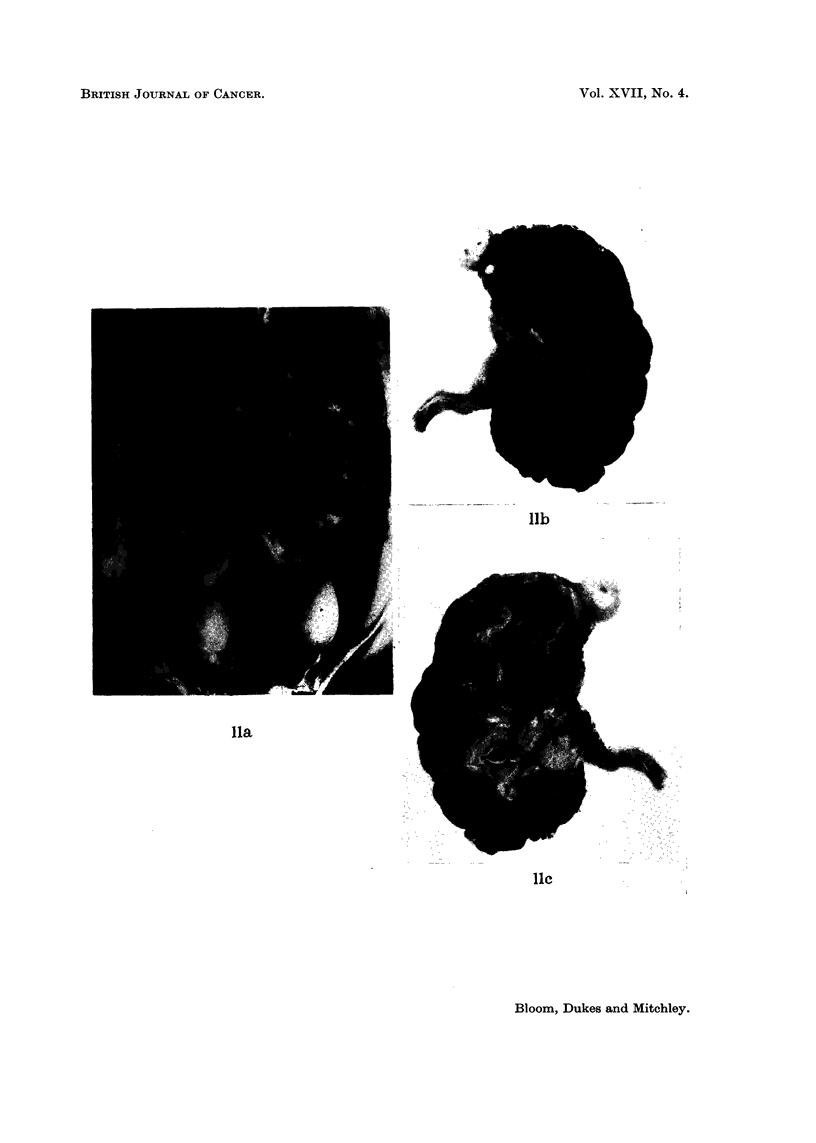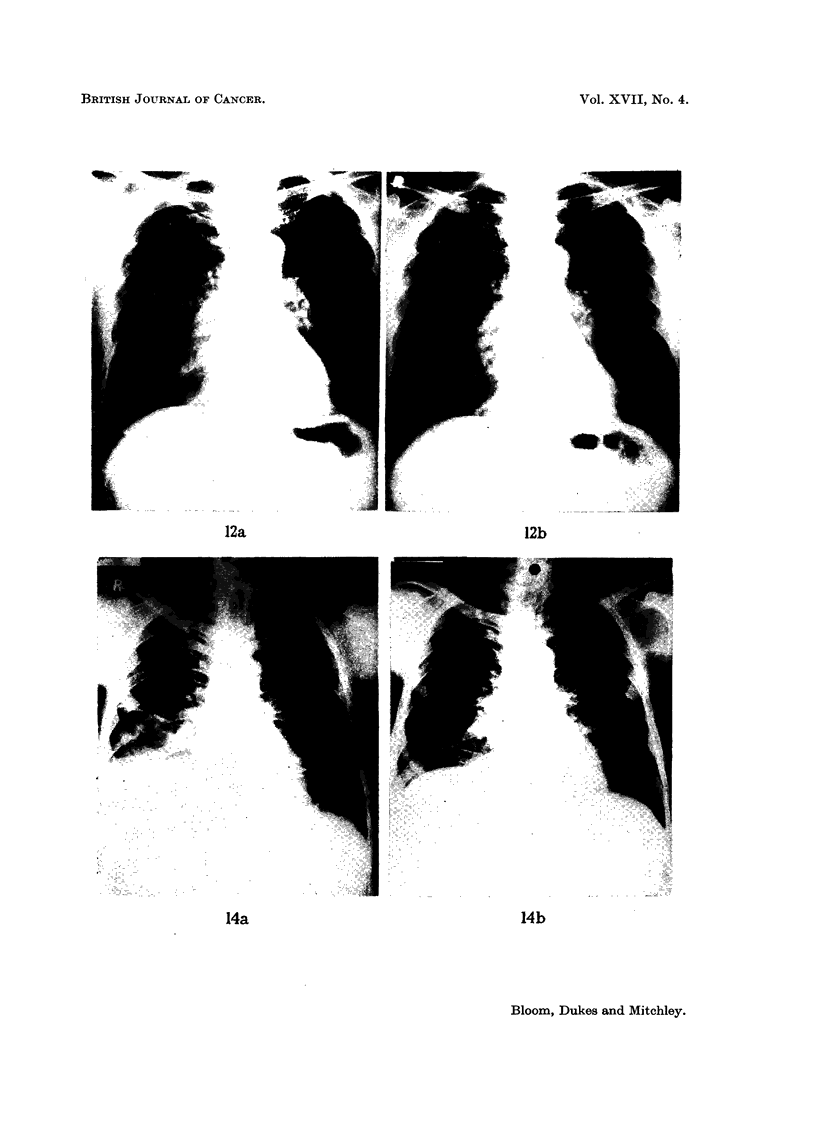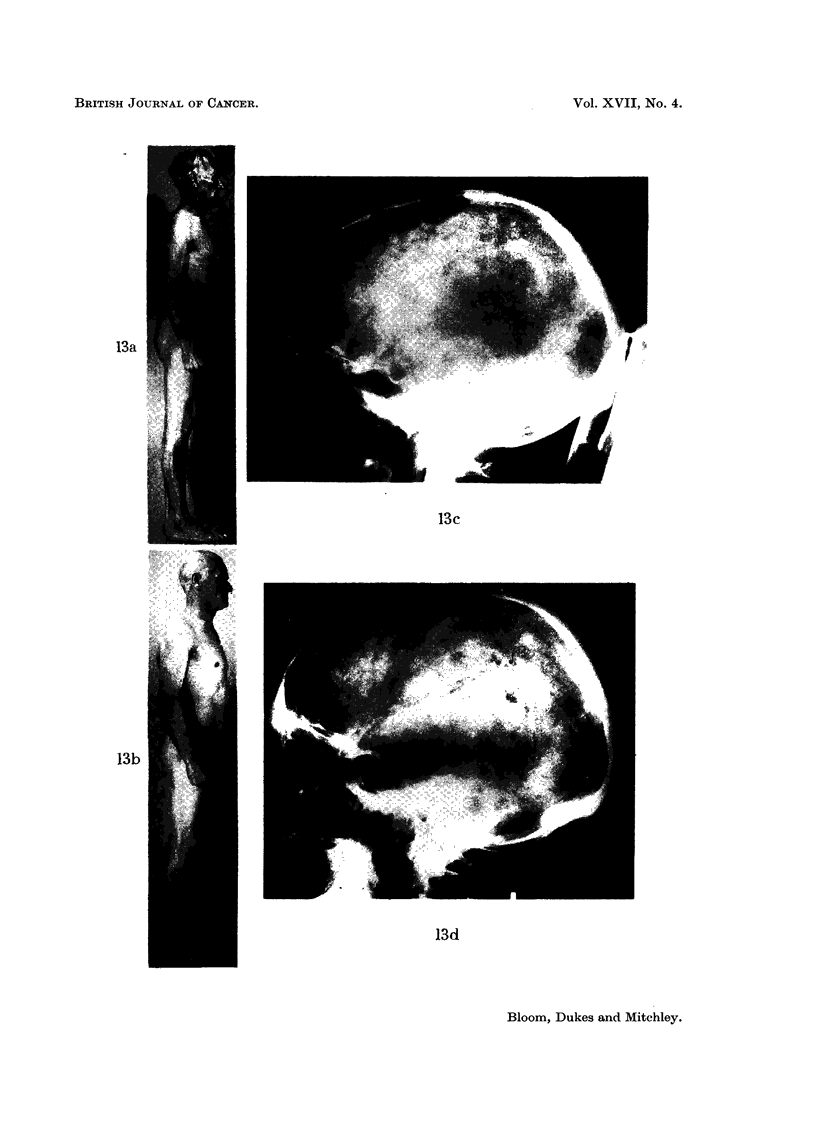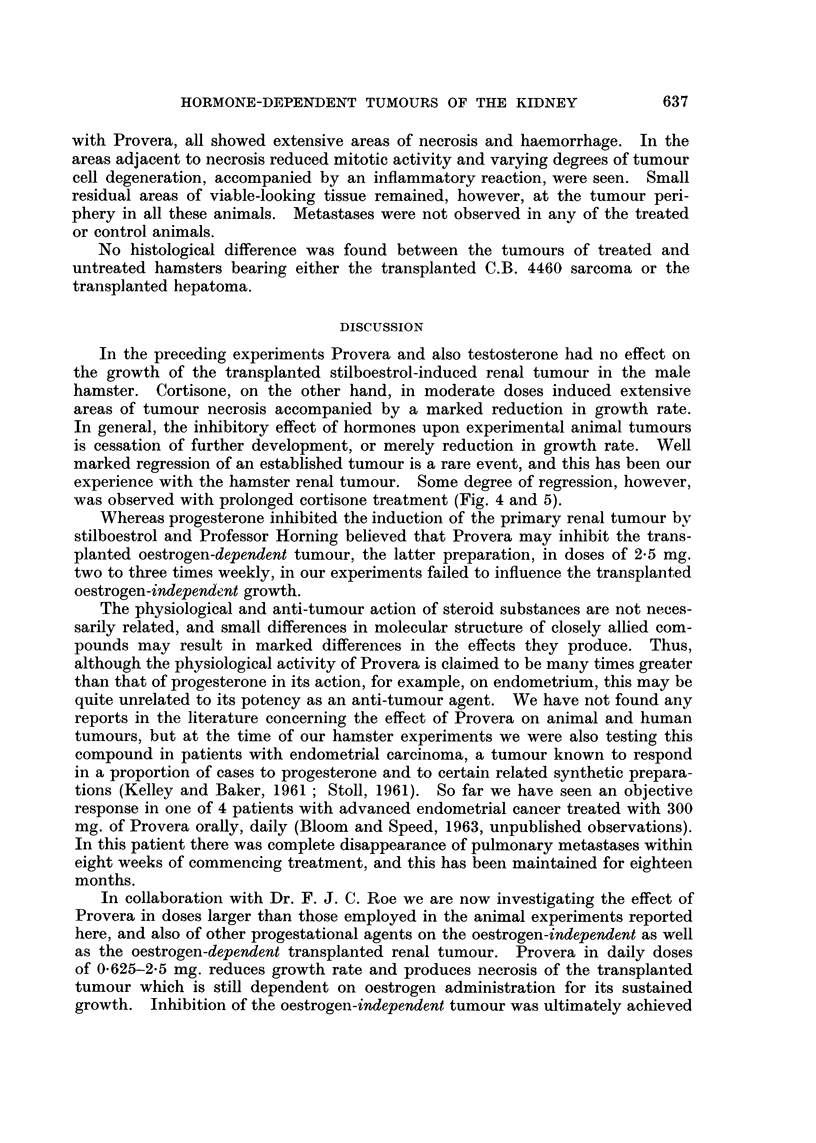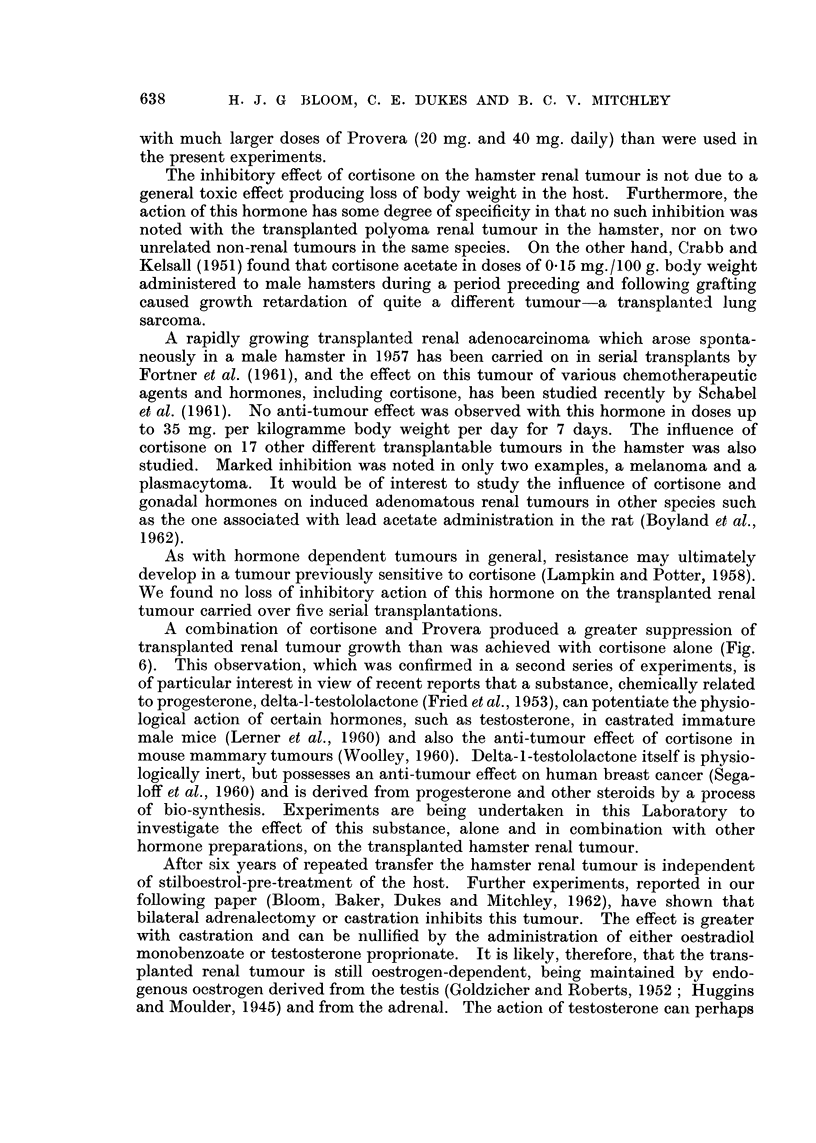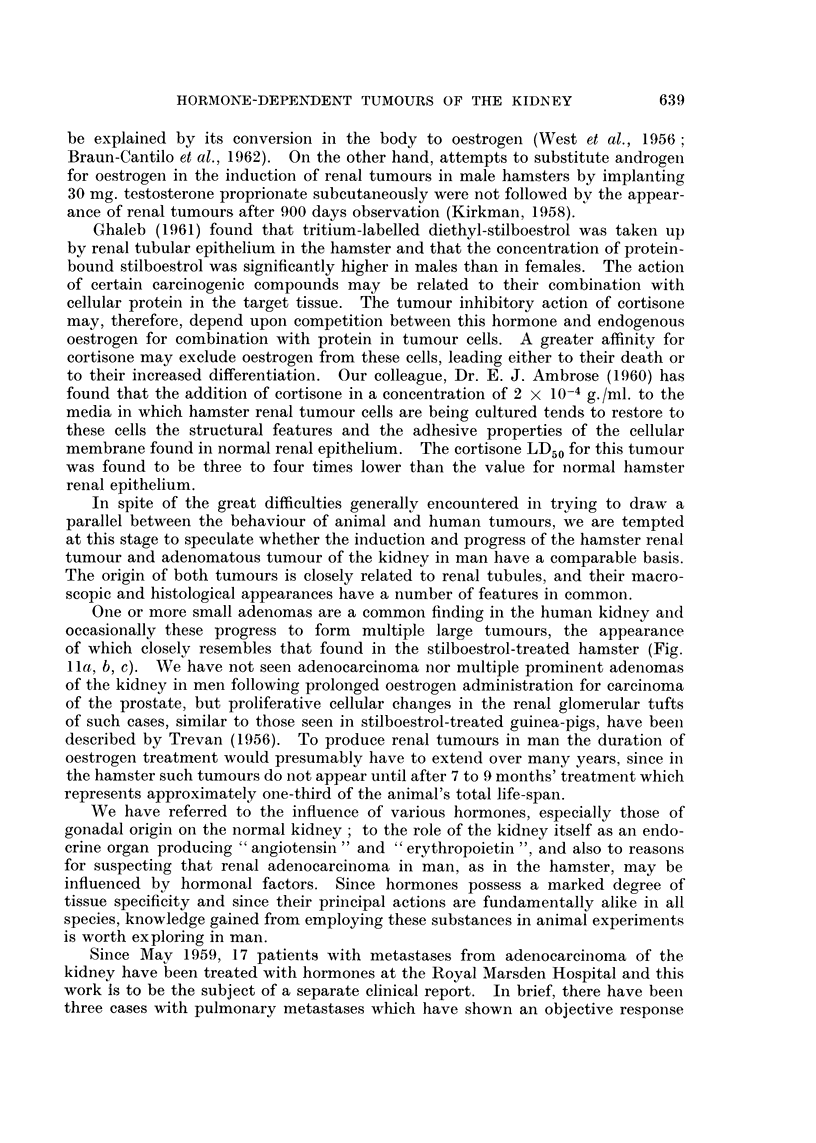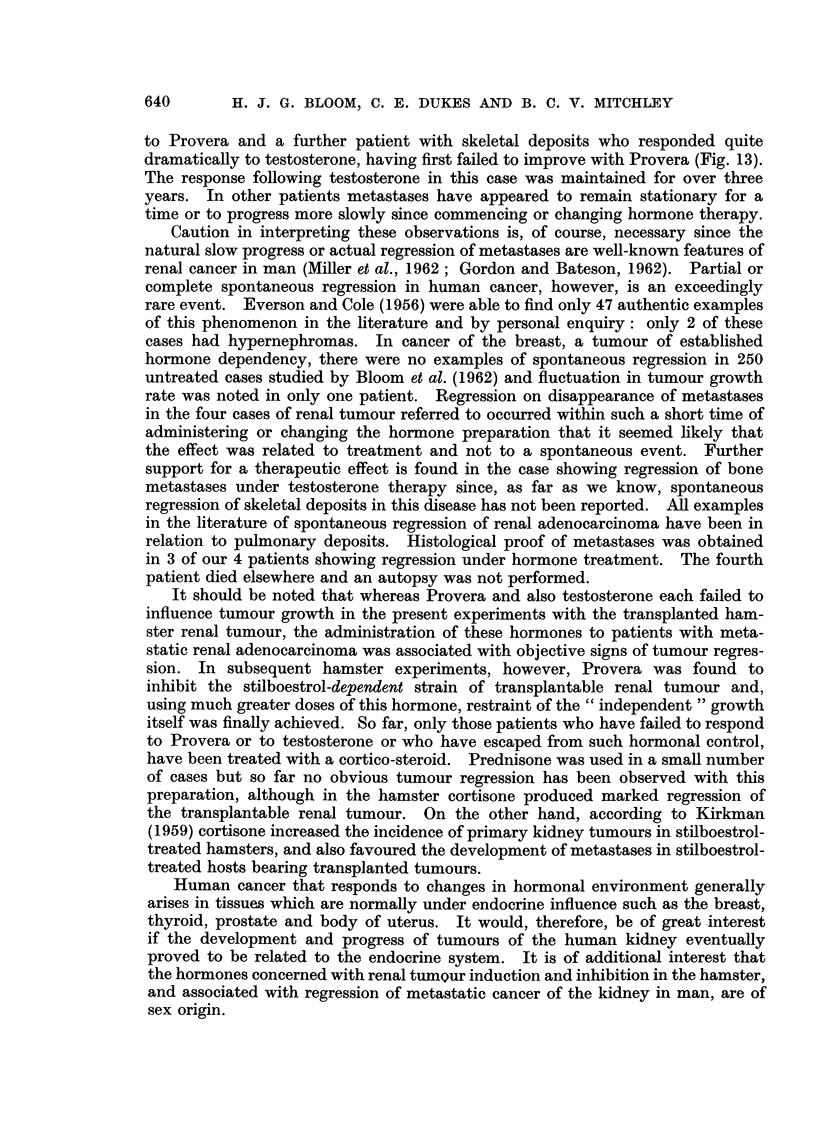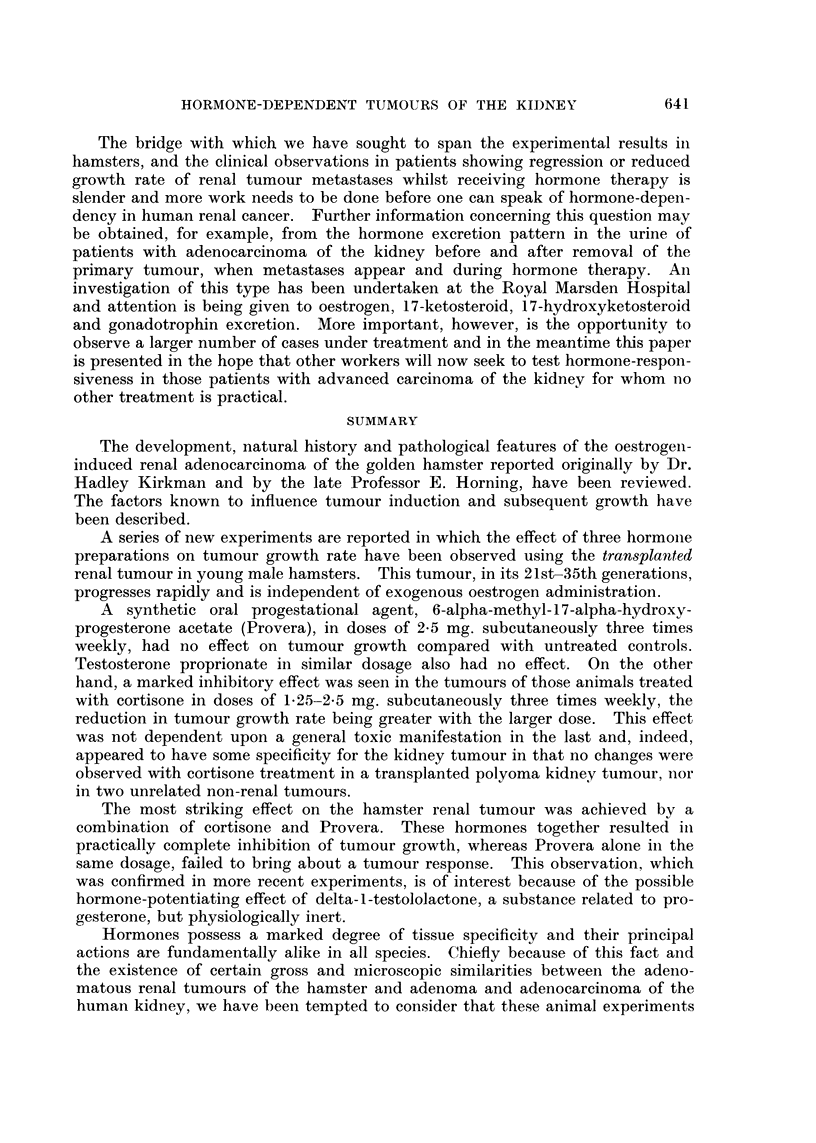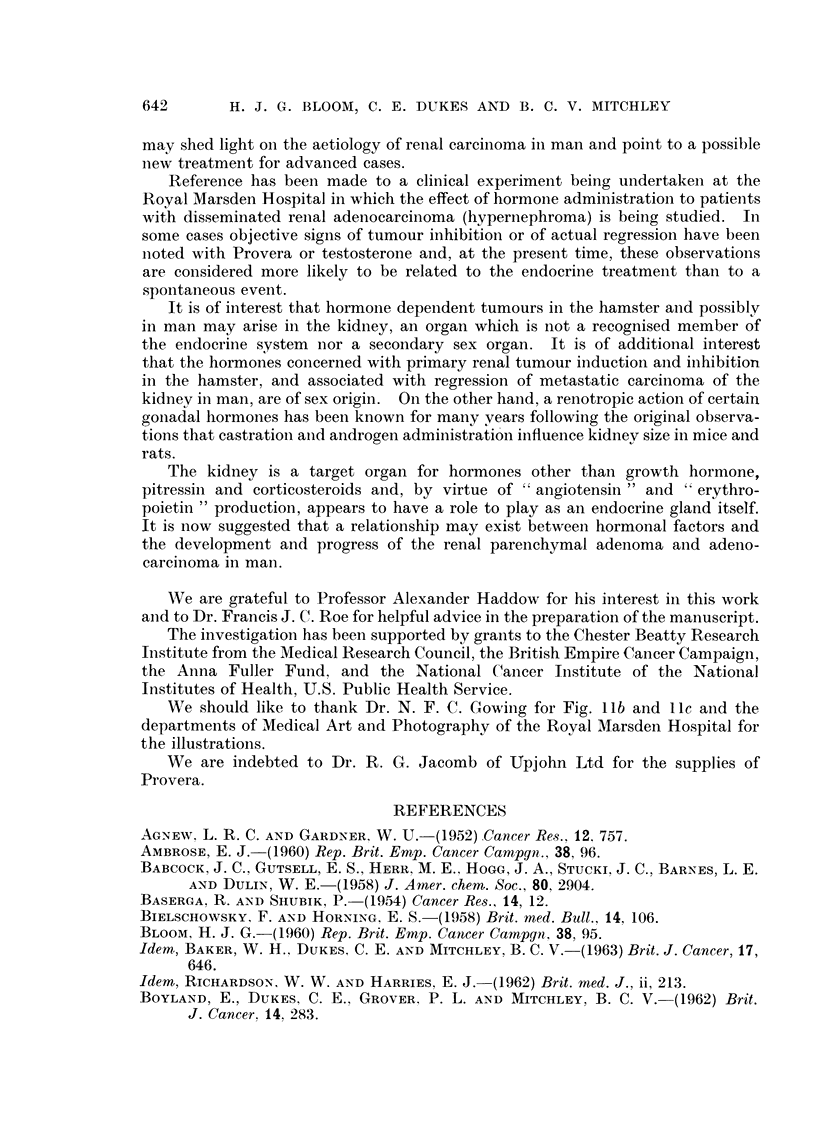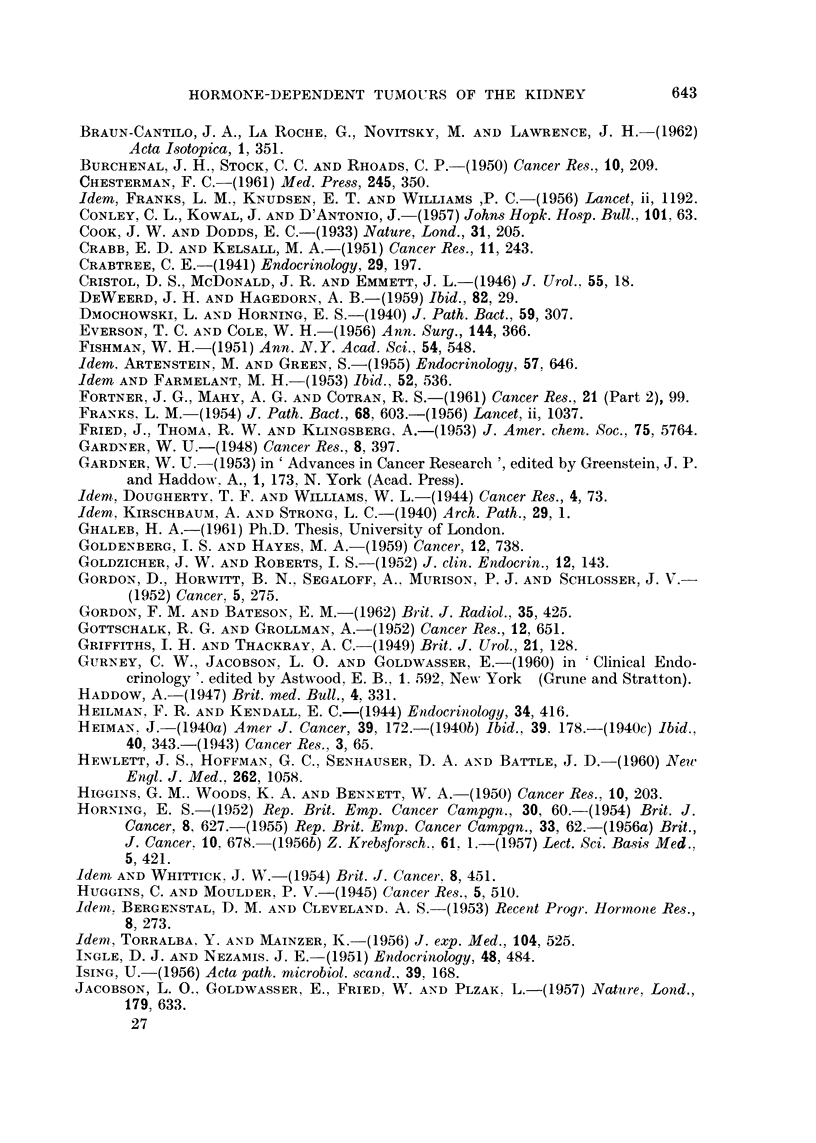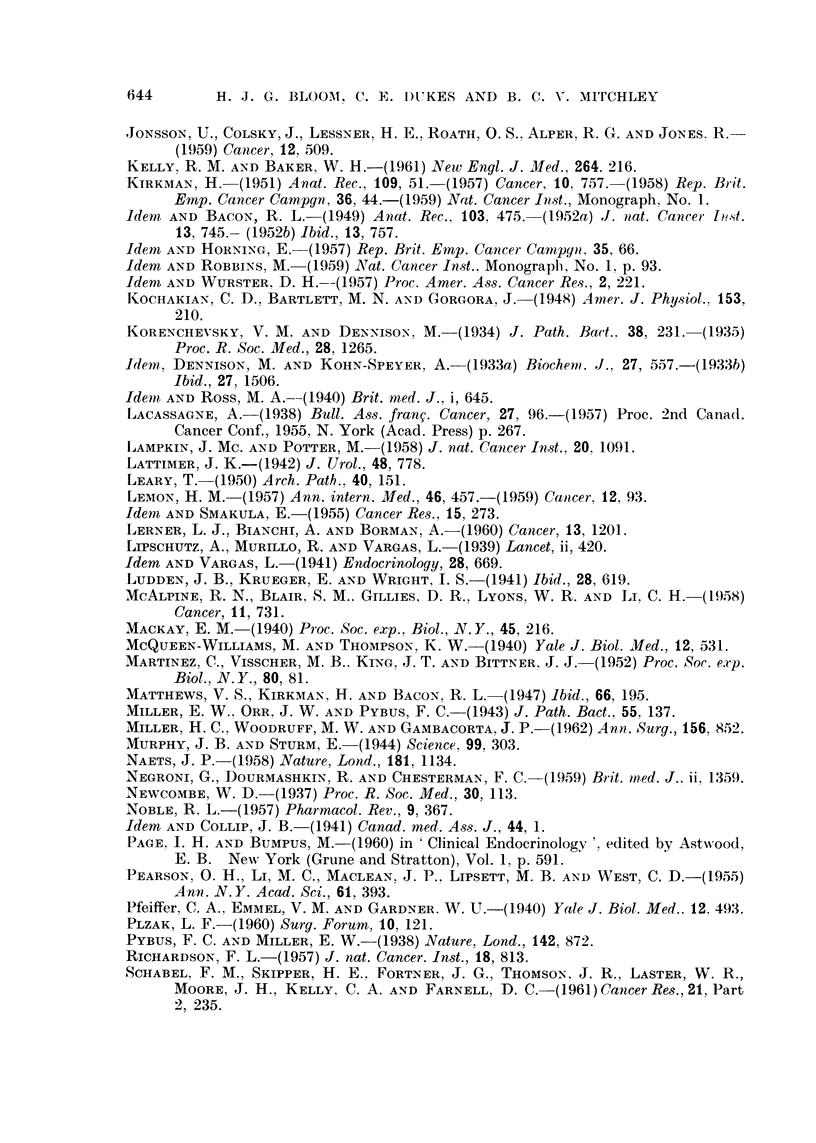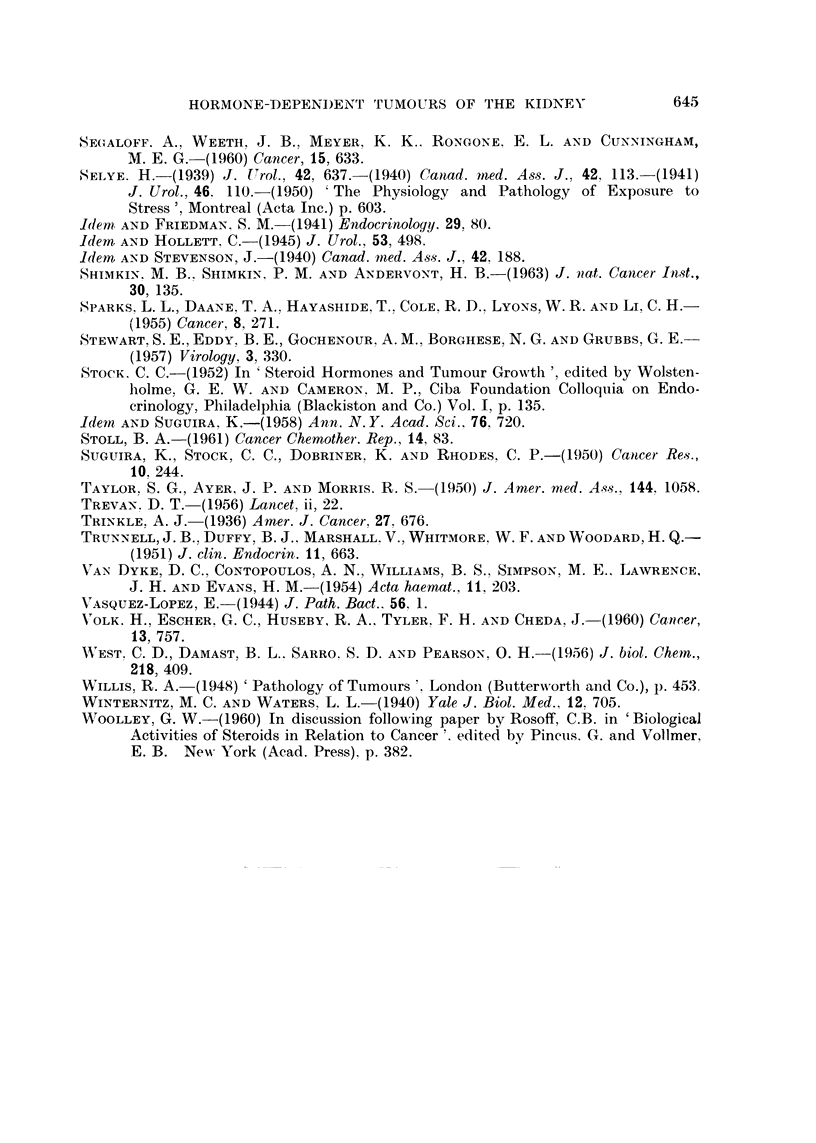# Hormone-Dependent Tumours of the Kidney

**DOI:** 10.1038/bjc.1963.81

**Published:** 1963-12

**Authors:** H. J. G. Bloom, C. E. Dukes, B. C. V. Mitchley

## Abstract

**Images:**


### HORMONE-DEPENDENT TUMOURS OF THE KIDNEY

### I. THE OESTROGEN-INDUCED RENAL TUMOUR OF THE SYRIAN HAMSTER. HORMONE TREATMENT AND POSSIBLE RELATIONSHIP TO CARCINOMA OF THE KIDNEY IN MAN

H. J. G. BLOOM, C. E. DUKES AND B. C. V. MITCHLEY

*From the Royal Marsden Hospital and the Chester Beatty Research Institute, Institute of Cancer Research, Royal Cancer Hospital, London, S.W.3*

Received for publication August 14, 1963

BY altering hormonal balance in experimental animals it has been possible to produce tumours of such organs as the pituitary, thyroid, adrenal, breast, ovary, uterus and testis (Bielschowsky and Horning, 1958; Gardner, 1948, 1953; Lacassagne, 1957; Noble, 1957). All these organs are either members of the endocrine system or else secondary sexual organs which are greatly influenced by this system. Only rarely have hormonal factors been associated with the development and subsequent behaviour of tumours in organs or tissues normally not under endocrine control. Lymphomas in mice are influenced by oestrogen, the incidence being increased by this hormone and reduced by ovariectomy or by androgen administration (Lacassagne, 1938; Dmochowski and Horning, 1940; Gardner *et al.*, 1940, 1944). The latent period for the development of spontaneous bone tumours in male mice is reduced by oestrogen administration and prolonged in females by ovariectomy (Pybus and Miller, 1938; Miller *et al.*, 1943). The incidence of hepatic tumours in this species is also reduced by oestrogen (Agnew and Gardner, 1952) and regression of osteogenic sarcoma and lymphosarcoma can be brought about by cortisone (Stock, 1952).

In 1944 Vasquez-Lopez noted the presence of a tumour in the kidney of a male Syrian golden hamster some 300 days after subcutaneous implantation of a stilboestrol pellet. Vasquez-Lopez regarded the renal lesion as a secondary deposit from either a tumour of the epididymis or the pituitary, both of which were also present. It was left to Matthews, Kirkman and Bacon (1947) to discover that the hamster possesses a peculiar susceptability to renal neoplasia in response to prolonged oestrogen administration. This observation was later confirmed and extended by Kirkman and Bacon (1949, 1952*a*, 1952*b*), Kirkman (1959), Horning (1952, 1954, 1956*a*, 1956*b*, 1957) and by Horning and Whittick (1954).

The hamster renal tumour is of special interest for three reasons. In the first instance it is a hormone-dependent tumour which has been readily induced in an organ which does not belong to the endocrine system and one which is not generally regarded as coming under the influence of the anterior pituitary gland, other than the general effects of its growth hormone (Horning, 1956*a*). Second, the hormones concerned with the induction (oestrogens) and prevention (testosterone and progesterone) of this tumour are of gonadal origin. Finally, of all observations concerning carcinogenesis in experimental animals those related to endocrine factors are perhaps the most likely to be of value in man, since the principal action of individual hormones is fundamentally alike in all species.

It is the purpose of this and the following paper (Bloom *et al.*, 1963) to report experiments concerning the influence of various hormone preparations and of endocrine ablation procedures on the growth of the transplanted hamster renal tumour. We will also refer to the influence of various hormones on the normal kidney and to the role of the kidney as an endocrine organ. Our goal is to draw attention to the possible application of experimental observations in the hamster to the endocrine treatment of renal adenocarcinoma in man. Earlier experimental work with the hamster tumour has been reported in a number of papers by Dr. Hadley Kirkman and his colleagues from Stanford University, and by the late Professor Eric Horning from this Laboratory, and also by both these authors in notes for the Annual Reports of the British Empire Cancer Campaign between 1952 and 1958. Since many pathologists and clinicians are unlikely to be familiar with the natural history and characteristics of the hamster kidney tumour it has been deemed worth while to review previous work on the subject before reporting our own observations in this field. Particular attention will be given to the monograph published by Kirkman (1959) which appeared soon after the present investigation had been initiated.

### I. REVIEW OF PREVIOUS WORK

Prolonged oestrogen administration to rodents may produce degenerative or proliferative lesions in the kidney. Such changes have been described in mice (Selye, 1939), rats (Pfeiffer *et al.*, 1940; Korenchevsky and Ross, 1940) and in guinea pigs (Chesterman *et al.*, 1956). The proliferative changes of the glomerular tuft reported in guinea pigs have also been described in men receiving stilboestrol for prostatic cancer (Trevan, 1956).

The Syrian hamster possesses a peculiar susceptability to oestrogen. In this animal, and only in the male, the predominant renal lesion produced by prolonged administration of stilboestrol is proliferative in type, eventually leading to the formation of adenomatous tumours. In the intact female such tumours are not found following oestrogen administration, the chief changes being amyloid infiltration and tubular atrophy (Matthews *et al.*, 1947).

The experiments carried out by Horning in this Laboratory have shown that, following subcutaneous implantation of a 20 mg. pellet of diethyl-stilboestrol in male hamsters 6–8 weeks of age, renal tumours develop and become palpable within 9–12 months in 70–80 per cent of treated animals. By renewing the pellet at three to four months tumour incidence can be increased to practically 100 per cent—the lesions are considerably larger, appear earlier and metastasize more readily.

After approximately seven months the tumours appear as small pale areas in the renal cortex beneath the capsule. As treatment continues they increase in size and number eventually form multiple, large bilateral tumours showing haemorrhagic, necrotic and cystic changes. Although well-defined the tumours are not truly encapsulated. After nine months the lesions are easily palpable in the flank of the living animal.

The earliest neoplastic change consists of minute clusters of abnormal cells in close proximity to the convoluted tubules. The tubular cells increase in size, proliferate and obliterate the luminae. Infiltration into the surrounding parenchyma occurs and, finally, the fully established tumour consists of compact masses of pale round cells. Abundant intracytoplasmic doubly refractile lipoid material is sometimes present producing a vacuolated appearance. Occasionally, tubule formation is present, but a papillary or pseudo-glandular appearance is more common.

*Histogenesis*

Horning and Whittick (1954) considered the hamster kidney tumour to be an adenocarcinoma arising from proximal convoluted renal tubules. Kirkman and Robbins (1959) agreed in the main with this view, but believed that the tumour was also partly of connective tissue origin. Areas of early cellular hyperplasia produced by prolonged oestrogen administration, although adjacent to renal tubules, not infrequently appear to be quite separate from them as if arising from inter-tubular cellular elements.

There is no clear-cut division on histological grounds between simple hyperplastic foci, adenoma and carcinoma in the renal parenchyma, all stages of tumour development being observed, often in the same specimen.

*Tumour spread and metastases*

After nine to ten months' treatment with stilboestrol the renal tumour is likely to have extended beyond the primary site. Generally speaking, spread of these tumours appears to be by direct surface implantation of detached tumour cells and is confined to the abdominal cavity. Nodules, plaques and sheets of tumour may be found on the inferior surface of the diaphragm and on the surface of the abdominal viscera. Kirkman and Robbins (1959) have reported distant metastases in cervical lymph nodes and in the lungs. On the other hand, Horning and Whittick (1954) did not observe distant metastases in any of their tumour-bearing animals.

*Tumour transplantation*

The presence of excess oestrogen is necessary for the induction and sustained growth of the hamster renal tumour, and also for its survival and further growth as a transplant. Thus, it is necessary to implant stilboestrol in prospective hosts three months before the tumour is grafted, if a successful take and subsequent growth is to be achieved. Initially, a latent period of seven to twelve months elapsed before the subcutaneous grafted tumours became palpable in the flank of 50 per cent of host animals (Horning, 1956*a*) but with repeated transfer the number of successful takes has increased and the latent interval become progressively shorter.

At the Chester Beatty Institute the transplantable renal tumour, after remaining dependent upon continuous oestrogen treatment of the host for nearly five years of serial grafting, eventually ceased to be dependent upon exogenous oestrogen administration. Kirkman and Horning (1957), whilst working together also in this Laboratory, reported successful takes of this tumour in 4 of 10 untreated male hamsters, but the tumours became palpable only after an average latent period of 11·5 months. With each succeeding generation, however, this interval has steadily decreased and now, after a period of nine years of repeated transfer, the present tumour (forty-fifth generation) becomes palpable in the flank of practically all animals within two to three weeks of transplantation.

Transplanted experimental tumours which are initially dependent on hormone pre-treatment of the host for their sustained growth and which subsequently acquire autonomy, generally arise in endocrine tissues or in secondary sexual organs. The behaviour of the transplanted hamster renal tumour, although arising from an organ which is not a recognized member of the endocrine system, has much in common with such lesions.

*Factors Influencing Induction and Growth Rate of the Hamster Renal Tumour*

1. *Species and sex*

Spontaneous renal tumours are extremely rare in untreated stock hamsters of either sex. They have not been observed in this Laboratory by Horning and by the present authors, nor in over 3800 autopsies carried out in Kirkman's department (Kirkman, 1957). However, a spontaneous renal adenocarcinoma arising in a male hamster in 1957 and carried on by serial transplantation has been reported recently by Fortner *et al.* (1961).

The hamster renal tumour with which we are concerned is readily induced by oestrogen only in males. In 268 male hamsters treated with various oestrogen preparations for 136–659 days renal tumours appeared in 74 per cent of animals (Kirkman, 1959). This tumour was successfully induced in 450 male hamsters by Horning (1956*b*), but the total number of animals treated is not known.

The renal epithelium of the female hamster does not readily undergo neoplastic change following oestrogen administration. Kirkman (1959) has seen only poorly developed tumours in one of 56 female hamsters following oestrogen administration for periods ranging from 241 to 487 days. Perhaps a more prolonged treatment with higher doses of oestrogen is necessary to induce tumours in the female. On the other hand, ovariectomy renders the female hamster susceptible to the development of this tumour (Kirkman, 1951 ; Kirkman and Wurster, 1957) which was found in 66 per cent of 41 castrated females treated with oestrogen (Kirkman, 1959).

Kirkman (1959) has also been able to produce renal tumours in female hamsters by manoeuvres other than castration. Thus, by commencing oestrogen administration within the first few days of life, or by treating new-born animals with testosterone proprionate before oestrogen administration, it has been possible to produce tumours in females in whom the ovaries have not been removed.

Tumours of the kidney have only rarely been induced by oestrogen in rodents other than the hamster. Richardson (1957) reported an incidence of 2 per cent among male mice treated with stilboestrol.

2. *Type of oestrogen*

Most of the work on the hamster renal tumour has been with diethyl-stilboestrol administered either by injection or as a subcutaneous implant. Oestrone and oestriol also produce renal tumours, but the latent period with these hormones is greater than with stilboestrol (Kirkman, 1959). In earlier experiments Kirkman and Bacon (1952*b*) failed to induce renal tumours in 12 hamsters with ethyl oestradiol, but later reported such tumours in 5 of 17 animals treated with this preparation (Kirkman, 1959).

3. *Dose of oestrogen and duration of treatment*

Kirkman and Bacon (1952*b*) found that 0·6 mg. of stilboestrol injected subcutaneously on alternate days for more than 250 days produced renal tumours in all of 11 animals, whereas the same amount given every tenth day for 400 days was quite ineffective. It is to be noted that in the latter case the total amount of oestrogen administered was only 24 mg. compared with 75 mg. in the more frequent treatment. Horning (1956*b*), who treated his hamsters with subcutaneous pellets of 20 mg. of stilboestrol, found that the incidence, size and spread of induced renal tumours was increased by a second 20 mg. pellet implanted 3 to 4 months after the first. By this means the number of successful transplants was also increased and the latent period shortened.

The duration of oestrogen treatment influences the induction of renal tumours. Thus, Kirkman and Bacon (1952*a*) found that in 100 animals treated for 250–600 days the tumour incidence was 97 per cent, whereas no such tumours developed among animals treated for less than 150 days. The observed difference, however, may have been due to the difference in total dose rather than to duration of exposure.

4. *Unilateral nephrectomy or ureterectomy*

Tumours appeared earlier and were larger in the remaining kidney of stilboestrol treated hamsters following unilateral nephrectomy (Horning, 1954), and in the ipsilateral kidney after unilateral ureterectomy (Ising, 1956). In the former case the mean duration of treatment necessary for tumour induction was 190 days compared with 286 days in unoperated animals. Horning (1954) suggested that the enhanced effect in his nephrectomised animals was due to a greater concentration of oestrogenic carcinogen in the remaining kidney, but a more likely explanation is that a kidney stimulated to hypertrophy is more sensitive to carcinogens than a kidney not subjected to this stress. Kirkman (1959) repeated Horning's experiment in 12 hamsters, but failed to show any difference in tumour size compared with 8 intact animals.

5. *Withdrawal of oestrogen*

When oestrogen administration is abruptly withdrawn regression of the primary renal tumour and of transplanted tumour tissue occurs. In such cases re-implantation of stilboestrol, even after 200 days, is followed by tumour regeneration and regrowth (Kirkman, 1959). There is some evidence from endocrine ablation experiments with replacement therapy described in our subsequent paper (Bloom *et al.*, 1963) which suggests that the transplanted tumour, when deprived of stilboestrol, may remain under the influence of endogenous oestrogen from such organs as the testes or adrenals. These glands may be responsible for maintaining tumour viability over long periods following withdrawal of administered oestrogen.

6. *Administration of chemical carcinogens*

(i) 20-*Methylcholanthrene*.—Implantation of this carcinogen in male hamsters treated with diethyl-stilboestrol reduced the incidence and growth rate of renal tumours. Small cortical tumours were present in only 20 per cent of animals treated in this way compared with multiple large tumours in over 80 per cent of animals treated solely with stilboestrol (Kirkman and Horning, 1957). Haddow (1947) has shown that chemical carcinogens may produce an inhibitory effect on tumour and general body growth.

(ii) 3,4-*Benzopyrene*.—Subcutaneous treatment with this agent induced two renal cortical tumours in 15 male hamsters (Horning, 1954). It is of interest that this carcinogen possesses weak oestrogenic activity (Cook and Dodds, 1933).

7. *Administration of various hormones*

(i) *Testosterone*.—Testosterone proprionate, in doses of up to 2·5 mg. subcutaneously once weekly, to young male hamsters implanted with 20 mg. of stilboestrol prevented the development of renal tumours (Horning, 1956*b*). Kirkman (1959) also found that the addition of testosterone to oestrogen inhibited the *induction* of this tumour, but noted a more rapid growth in the case of the *established* primary or the transplanted tumour.

(ii) *Progesterone*.—Horning (personal communication, 1959) had observed a possible inhibitory effect on the transplanted oestrogen dependent renal tumour with a progestational agent, 6-alpha-methyl-17-alpha-hydroxyprogesterone acetate (Provera) (Babcock *et al.*, 1958). Later in the same year Kirkman (1959) reported that renal tumours failed to develop in 11 intact male hamsters treated with combined implants of stilboestrol and progesterone for 293–452 days. In 7 other stilboestrol-treated animals the growth rate of transplanted renal tumour tissue was reduced by progesterone administration.

(iii) *Corticosteroids* : (a) *Deoxycorticosterone acetate*.—This hormone implanted in 11 stilboestrol-treated male hamsters inhibited the induction of renal tumours, except in one animal which showed microscopic nodules in the kidney. In 9 oestrogen-treated hosts bearing transplanted tumours deoxycorticosterone acetate brought about some reduction in growth rate of the graft compared with stilboestrol treated controls (Kirkman, 1959).

(b) *Cortisone*.—In 8 oestrogen-treated male hamsters bearing transplants of oestrogen-induced renal tumours Kirkman (1959) found that the addition of cortisone to stilboestrol appeared to increase the incidence of primary renal hamster tumours and of metastases.

To summarise : testosterone, progesterone or deoxycorticosterone acetate when administered to stilboestrol-treated male hamsters inhibited the induction of renal tumours. In the case of the transplanted tumour, progesterone and deoxycorticosterone acetate each reduced growth rate whereas testosterone appeared to accelerate it. This influence of sex hormones on a tumour of renal origin is of great interest, the kidney not being a recognized member of the endocrine system not a secondary sex organ.

### *Mechanism of Tumour Induction by Stilboestrol in the Hamster Kidney*

The mechanism of renal tumour induction by oestrogens is unknown. Prolonged administration of stilboestrol to the hamster also produced tumours of the pituitary pars intermedia in 65 per cent of animals (Horning, 1956*b*). At one time Horning (1955) planned to study the effect of pituitary ablation on the development of the oestrogen-induced renal tumour. Kirkman (1957) stated without giving details, that renal tumours could be induced by oestrogen in hypophysectomised animals and that they could be successfully transplanted in oestrogen-treated hypophysectomised hosts. This suggests that oestrogen may act directly on renal tissue and that an indirect action via the pituitary gland is unlikely. More recently, Kirkman (1959) referred to 4 hypophysectomised hamsters treated with stilboestrol, all of which developed bilateral renal tumours.

Ghaleb (1961), working in this Institute, with tritium-labelled diethyl-stilboestrol, found further evidence for a direct carcinogenic action of oestrogen on the hamster renal epithelium. The tracer hormone was taken up and bound to renal cellular proteins. In view of the sex-linked liability to develop renal tumours it was interesting to find that the renal epithelium of the male hamster concentrated twice the amount of labelled oestrogen compared with that of the female. Ghaleb suggested a possible relationship between renal carcinogenesis in the hamster and the binding of stilboestrol to kidney cellular proteins. On the other hand, Kirkman (1959) assumed that no fundamental difference existed between kidney tissue in male and female hamsters with regard to stilboestrol carcinogenesis, since tumours were found in fragments of renal tissue which had been taken from new-born male or female donors and transplanted into stilboestrol-treated male hosts.

### II. PRESENT INVESTIGATION

Small renal adenomas are a common finding at routine human autopsy, especially in men over age forty with nephrosclerosis. In such cases it is possible that cholesterol is a stimulant to tubular proliferation. Leary (1950) has described a sequence of events from the deposition of cholesterol ester crystals in renal epithelial cells to the formation of adenomatous tumours. There is no clear-cut division on histological grounds between adenoma and adenocarcinoma of low grade malignancy in the human kidney (Cristol *et al.*, 1946 ; Willis, 1948 ; Griffiths and Thackray, 1949), all gradations between a benign lesion and a carcinoma of high malignancy being seen. It is likely that at least some carcinomas of the kidney in man arise from these early adenomatous lesions (Trinkle, 1936 ; Newcombe, 1937) : the small foci seen at routine autopsy may represent latent or pre-invasive malignant tumours, a position comparable to that described in the prostate by Franks (1954 ; 1956) and possibly in other sites such as thyroid, breast and adrenal. The cause of latency in such tumours is unknown, but it is likely that hormonal environment in the host is involved.

The natural history of adenocarcinoma of the human kidney also suggests the possibility of this tumour being under hormonal influence. Thus, the very slow progress of some primary and metastatic tumours, the long interval which may occur between nephrectomy and the appearance of distant metastases, the examples of spontaneous regression and those rare cases of prolonged survival or apparent cure following removal of a solitary deposit suggest that in such cases the tumour is not completely autonomous.

### *Hormones and the Normal Kidney*

In view of the observations concerning the oestrogen-induced renal cortical tumour in the hamster and the suggestion that adenomatous tumours of the human kidney may be influenced by the endocrine system, it is relevant to consider possible effects of this system on the *normal* kidney with special reference to hormones of gonadal origin.

The kidney is a target organ for posterior pituitary anti-diuretic hormone and for adrenal cortico-steroids which control water and salt excretion respectively. The anterior pituitary influences renal structure, probably by virtue of its growth hormone. Thus, in experimental animals hypophysectomy causes renal atrophy (Selye, 1941) and prevents the compensatory hypertrophy of the remaining kidney following unilateral nephrectomy (Winternitz and Waters, 1940 ; McQueen-Williams and Thompson, 1940). Administration of crude anterior pituitary extracts to hypophysectomized rats restores kidney weight (Selye, 1941) and pre-prevents the atrophy which follows unilateral ureteric ligation (Selye and Hollett, 1945).

It has been known for thirty years that gonadal hormones influence the kidney in certain experimental animals. Thus, gonadectomy reduces kidney weight in male rats, and androgens induce renal hypertrophy in normal and ovariectomized female rats and in castrated males (Korenchevsky *et al.*, 1933*a*, 1933*b* ; Korenchevsky and Dennison, 1934, 1935 ; Korenchevsky and Ross, 1940). Selye (1939) found that androgens enlarge the kidneys in both intact and castrated mice of both sexes. These hormones increase the compensatory hypertrophy of the remaining kidney following unilateral nephrectomy (Mackay, 1940 ; Lattimer, 1942) and protect the kidney from atrophy caused by ureteric ligation (Selye and Friedman, 1941). Oestrogens, on the other hand, were found to produce essentially degenerative changes in the rat and mouse kidney (Korenchevsky and Ross, 1940 : Selye, 1939). Ludden *et al.* (1941) found an increase in kidney weight in rats treated with oestradiol benzoate, but this was attributed to water retention in the renal tissue. On the other hand, Shimkin *et al* (1963) have recently reported experiments in male mice of the ACLB and C3H strains in which oestrogen produced a rapid and sharp drop in kidney weight. There was some evidence that a direct hormonal effect on the kidney itself was responsible for these changes. Histological examination did not reveal any abnormality, and it was postulated that the weight increase was related to a disturbed electrolyte-water balance.

The chief histological changes produced in the kidney by androgens are hypertrophy of the convoluted tubules and of the parietal cells of Bowman's capsule (Selye, 1939). These changes are essentially an exaggeration of the normal sex difference in the renal architecture of most strains of mice. The changes in Bowman's capsule may also be induced by pregnancy (Crabtree, 1941).

Progesterone also caused an increase in kidney weight when administered to mice and rats of either sex (Selye and Stevenson, 1940 ; Selye, 1940). This effect was antagonistic to the weight-depressant action of oestrogens.

The renotropic action of testosterone and of progesterone is probably a direct one since these hormones exert kidney-stimulating effect in the hypophysectomized rat (Selye, 1941).

Fishman (1951) and Fishman and Farmelant (1953) have shown that the concentration of glucoronidase in the mouse kidney is selectively increased by testosterone treatment. This action appears to be specific for androgens as distinct from oestrogens, progesterones and cortico-steroids and is nullified by stilboestrol (Fishman *et al.*, 1955).

Although the principal effect of various hormones is essentially the same in all species the changes produced by gonadal hormones on the kidney are not comparable in all experimental animals. Thus, in contrast to the findings in mice and rats neither castration nor androgen administration produced an appreciable increase in kidney weight in the hamster, although the concentration of renal enzymes in this animal was altered by these procedures (Kochakian *et al.*, 1948).

The kidney itself appears to fulfil the role of an endocrine organ. It is the site of production of " angiotensin " which is regarded as a renal hormone concerned with the control of blood pressure and blood distribution (Page and Bumpus, 1960). More recently, investigations have indicated that the kidney may also be involved in the control of red blood cell formation, and the site of production of " erythropoietin " (Jacobson *et al.*, 1957 ; Naets, 1958 ; Gurney *et al.*, 1960 ; Plzak, 1960). The occasional association of adenocarcinoma of the kidney with polycythaemia (Conley *et al.*, 1957 ; DeWeerd and Hagedorn, 1959) provides a possible link between tumours arising from the human renal cortex and hormone activity. With removal of the tumour the blood picture returns to normal: with the development of metastases the polycythaemic picture may be re-established. Hewlett *et al.* (1960) have found increased amounts of erythropoietin in extracts prepared from renal adenocarcinoma in polycythaemic patients.

Erythropoietin production by the kidney appears to be under pituitary control, hypophysectomy in the experimental animal being followed by severe anaemia and interference with the erythropoietic response to anoxia (Van Dyke *et al.*, 1954).

All these facts suggest that the relationship between hormones and the normal kidney is much greater than is perhaps generally appreciated. The question we seek to answer is whether this endocrine influence extends to tumours of renal origin, to adenocarcinoma of the kidney in man. This concept, together with the observations on renal tumour production by stilboestrol in hamsters prompted one of us, early in 1959, to consider hormone therapy for patients with metastatic adenocarcinoma of the kidney (Bloom, 1960). At that time Professor Horning had commenced some preliminary observations on the effect of the progestational agent, Provera, on hamster renal tumours. Although his results were inconclusive he thought that this substance might exert some degree of inhibition on the transplanted *dependent* renal tumour.

It was decided to try Provera in human renal cancer. In May 1959 a very ill woman aged 28 with abdominal metastases was treated with this preparation, but no objective response was observed and the patient died within three months. A second patient, a man of 64 with pulmonary and skeletal metastases, commenced Provera in August 1959 and within five weeks there were radiological signs of regression of the pulmonary metastases. Since then several other cases have been treated with Provera and also other hormones, and this experience will form the subject of a separate clinical report in collaboration with Mr. D. M. Wallace.

In October 1959 H.J.G.B. hoped to collaborate with Horning in some further animal experiments to test various hormones against the hamster tumour with the aim of applying such observations to the treatment of human renal adenocarcinoma, but Horning died on November 14th before the meeting to plan these experiments could take place. In January 1960 the present team came together and, whilst the clinical observations continued, the following animal experiments were undertaken.

### EXPERIMENTS

Since it requires some nine months to induce palpable renal tumours in the hamster with stilboestrol it was decided to investigate the influence of hormone administration on the *transplanted* tumour which was available for immediate use. This tumour, in its twentieth generation and independent of stilboestrol administration to the host, gave a high percentage of successful takes and formed a palpable nodule in the subcutaneous tissue, 8–10 mm. in diameter, within 2–3 weeks of transplantation.

### 1. *Hormones Studied*

(a) *Provera*

Compared with oestrogens and androgens, progesterone and progesterone-like substances have received little attention in relation to experimental and human tumour growth. The reports available regarding the action of progesterone on tumour development are often contradictory, but in general this hormone has an antagonistic effect on experimental oestrogen-induced tumours such as mammary growths in rats, (Noble and Collip, 1941 ; Heiman, 1943) and abdominal fibromyomas in guinea pigs (Lipschutz *et al.*, 1939).

It is interesting to recall that Selye and Stevenson (1940) and Selye (1941) found that progesterone had a direct effect on the normal kidney of the mouse and rat. Kirkman (1959) reported that this hormone inhibited the development of the adenomatous renal tumour in oestrogen-treated male hamsters.

At the time of planning our experiments there were few reports concerning the use of progesterones for human cancer. These indicated a possible effect in carcinoma of the prostate (Trunnell *et al.*, 1951) and breast (Gorden *et al.*, 1952). Further reports subsequently appeared concerning progesterones in the treatment of breast cancer (Goldenberg and Hayes, 1959 ; Volk *et al.*, 1960 ; Jonsson *et al.*, 1959) and carcinoma of the endometrium (Kelly and Baker, 1961), and these are of special interest in view of the known relationship of these tumours to oestrogen.

Provera, the trade name for 6-alpha-methyl-17-alpha-hydroxyprogesterone acetate (Babcock *et al.*, 1958), was the progestational agent chosen for both our hamster experiments and clinical studies, primarily because of the preliminary observation made by Horning (personal communication) in a small number of animals that this substance appeared to inhibit the transplanted oestrogen-*dependent* renal tumour. This preparation also appeared to be the most active progestational agent known, possessing a high degree of oral efficiency with no or very little androgenic and oestrogenic properties. It is considered to be 50–60 times more active than ordinary progesterone on subcutaneous administration, and 100–300 times more active than ethisterone when taken orally (Babcock *et al.*, 1958).

Provera has a very low toxicity even in high dosage. Single doses of up to 10,000 mg./kg. orally, and repeated doses of 30 mg./kg. daily for 190 days failed to produce any toxic effects in rats. The $LD_{50}$ single dose intravenously for mice was 376 mg./kg. (Upjohn Ltd., personal communication). In more recent experiments in this laboratory in collaboration with Dr. F. J. C. Roe, Provera was administered subcutaneously in doses of 20–40 mg. daily for 27 days to hamsters weighing 90–110 g. These animals remained generally well, but lost an average

7 per cent of their original weight with the lower dose and 20 per cent with the higher dose.

(b) *Testosterone*

Androgens have also been employed as antagonists to the neoplastic properties of oestrogen. Thus, in the mammary fibroadenoma of mice and rats testosterone decreases the number of successful transplants and reduces growth rate of established lesions (Heiman, 1940*a*, 1940*b*, 1940*c*, 1943 ; Huggins *et al.* 1956). Testosterone proprionate inhibits the development of oestrogen-induced uterine and abdominal fibroids in the guinea-pig (Lipschutz and Vargas, 1941) and opposes the leukaemic action of oestrogen in mice (Gardner *et al.*, 1944). Oestrogens have a role in experimental mammary carcinogenesis and in human breast cancer, and in both these fields testosterone has an inhibitory action.

We have already referred to the fact that androgens may act on the normal kidney of certain experimental animals, especially the mouse and rat, leading to an increase in weight of this organ due to hypertrophy and hyperplasia of the convoluted tubules.

Horning (1956*b*) in this Laboratory found that testosterone proprionate inhibited induction of the hamster renal tumour by stilboestrol. Kirkman (1959) confirmed this finding with the induced primary tumour, but observed a possible stimulating effect in the case of the oestrogen-*dependent* transplant.

(c) *Cortisone*

In recent years many observations have been made on the effect of cortisone and related compounds on the growth and dissemination of spontaneous and transplanted tumours in various laboratory animals.

Transplanted malignant lymphomas including leukaemic cells are markedly inhibited by cortisone in the mouse and rat (Heilman and Kendall, 1944 ; Murphy and Sturm, 1944 ; Burchenal *et al.*, 1950 ; Ingle and Nezamis, 1951 ; Lampkin and Potter, 1958). A less marked effect with large doses of this hormone has been reported in certain non-lymphomatous transplanted and spontaneous tumours (Stock and Suguira, 1958 ; Higgins *et al.*, 1950 ; Suguira *et al.*, 1950 ; Gottschalk and Grollman, 1952 ; Baserga and Shubik, 1954 ; Sparks *et al.*, 1955 ; MacAlpine *et al.*, 1958).

With large doses of cortisone it is possible to reduce the growth rate and induce areas of massive necrosis in certain experimental tumours. The extent of tumour destruction varies from 25 to 90 per cent : complete destruction is never seen. The doses of cortisone required to bring about marked tumour changes also induce inflammatory and degenerative lesions in vital organs such as the lungs, liver and kidney. The treated animals become ill and die, not from the tumour but from hormone treatment. The addition of 0·1 per cent terramycin to the drinking water reduces the side-effects of cortisone without interfering with its tumour inhibitory action (Martinez *et al.*, 1952).

Cortisone has been used in the treatment of a wide variety of malignant tumours, but it is only in patients with acute leukaemia, lymphosarcoma and chronic lymphatic leukaemia that temporary regression is to be expected with this hormone. Occasionally, objective signs of improvement may occur in breast cancer (Lemon, 1957, 1959 ; Pearson *et al.*, 1955) and in prostatic cancer (Huggins *et al.*, 1953 ; Taylor *et al.*, 1950).

Tumour inhibition by cortisone has been reported in the hamster by Lemon and Smakula (1955) who studied a transplanted methylcholanthrene-induced sarcoma, and by Crabb and Kelsall (1951) in a transplanted mixed cell sarcoma of the lung. The doses of cortisone employed by Lemon and Smakula (1955) were also toxic for the host. Kirkman (1957) mentioned that deoxycorticosterone prevented renal tumour induction by oestrogen in male hamsters and that cortisone had no such effect. In a later paper Kirkman (1959) reported inhibition of renal tumour induction in 10 of 11 stilboestrol-treated animals using deoxycorticosterone as a 20 mg. subcutaneous implant. This hormone also reduced growth rate of the transplanted oestrogen-dependent tumour in 7 animals studied. Cortisone, on the other hand, appeared to increase the incidence of primary renal tumours and of metastases in stilboestrol-treated hamsters already bearing renal tumour transplants.

The structure and function of the normal kidney are influenced by adrenal corticosteroids. Moderate doses of these steroids in, for example, the rat produce renal hypertrophy (Selye, 1940 ; Ludden *et al.*, 1941) whilst toxic doses cause marked degenerative changes (Selye, 1950).

### (2) *Method*

The present experiments were conducted with male Syrian golden hamsters aged 12–16 weeks and weighing 90–110 g. which were bred in the laboratories of the Chester Beatty Research Institute. The 21st to 32nd generations of transplanted renal tumour, independent of oestrogen administration, were employed. Under general ether anaesthesia a fragment of tumour approximately 5 mm. in diameter was implanted subcutaneously by trocar into the animals' flank. In two to three weeks the tumour became palpable as a nodule some 8–10 mm. in diameter. Tumour size was determined daily by careful caliper measurement and expressed as the sum of two diameters. In the treated animals hormone administration was commenced three to four days after the tumour was easily palpable. Each hormone was given subcutaneously and usually three times weekly. The doses employed were well above physiological levels, being based on quantities in excess of those used for tumour treatment in man.

The hamsters were kept five in a cage, fed on a routine diet consisting of maize, sun-flower seeds, rat cake and peanuts, and maintained at room temperature. The animals were all killed after a period of observation or, in the case of the more prolonged experiments, only when the enlarging tumour became a burden to the host, or if the animal became ill from treatment. Post-mortem examinations were performed and tumour tissue and relevant organs taken for histological study.

### (3) *Procedures*

*Experiment I*

The following animal groups were studied.

*Group* 1. 6 control animals (transplanted tumours untreated with hormones).

*Group* 2. Provera (Upjohn Ltd.), 2·5 mg. twice weekly subcutaneously to 6 animals from day 20 following tumour transplantation.

*Group* 3. Cortisone (Rousell), 2·5 mg. daily subcutaneously Monday to Friday each week to 6 animals from day 20.

*Group* 4. Testosterone proprionate (Schering) 2·5 mg. twice weekly subcutaneously to 6 animals from day 20.

*Results.*—Tumour grafts were successful in all 24 animals. By day 30 following transplantation it was evident that the tumours of the animals receiving Provera or testosterone were comparable to the control tumours in the untreated animals. On the other hand, marked inhibition of tumour growth occurred in all animals treated with cortisone (Fig. 1–3). In one of the animals the tumour, after becom-

Transplanted Independent Renal Tumour in Male Hamsters – (Experiment I)

EFFECT OF TESTOSTERONE, PROVERA AND CORTISONE

FIG. 1.—Tumours not affected by testosterone or Provera but marked inhibition with cortisone treatment.

ing palpable, disappeared by day 21 and failed to reappear by day 41 : actual tumour regression was not observed in the remaining 5 animals treated with this hormone. The hamsters receiving cortisone became ill and two died on day 41 : the remaining 4 animals in this group were killed between day 43 and 49. The control animals and those receiving testosterone remained generally well and were destroyed only when their tumours became a burden between day 42 and 49.

On day 42 the 6 animals receiving Provera were divided into two groups. In 3 animals, bearing the largest tumours, Provera was replaced by cortisone

FIG. 2.—Range of tumour growth rate in 6 untreated hamsters. Growth curves lie within shaded area.

to see whether a well-established tumour could be influenced by this hormone. The remaining 3 animals continued on Provera as controls. In view of the toxic effects noted with 2·5 mg. cortisone daily this dose was given only three times weekly. The tumours in the Provera treated hamsters continued to grow well, but in the animals receiving cortisone there was marked tumour inhibition and some regression took place (Fig. 4). On the reduced dosage of cortisone, toxicity was less marked and 2 animals were allowed to continue to day 58 and one to day 87.

Transplanted Independent Renal Tumour in Male Hamsters – (Experiment I)

EFFECT OF CORTISONE IN 6 ANIMALS

(2·5 mg. x 5/week)

FIG. 3.—Range of tumour growth rate in 5 hamsters treated with cortisone. Growth curves lie within shaded area.

*Experiment* II

*Group* 1. 6 control animals (transplanted tumours untreated with hormones).

*Group* 2. Cortisone, 2·5 mg. three times weekly to 6 animals from day 21 when the transplanted tumours were palpable.

*Group* 3. Cortisone, 1·25 mg. three times weekly to 6 animals from day 21 when the tumours were palpable.

FIG. 4.—Progressive tumour growth in 6 animals treated with Provera. Inhibition and some regression in well-established neoplasms when Provera was replaced by cortisone administration in 3 animals.

*Group* 4. Cortisone, 2·5 mg. three times weekly to 6 animals from day 28 when the tumours were well-established and of moderate size.

*Results.*—The tumour grafts were successful in all 24 animals and those in the control group grew rapidly (group 1 in Fig. 5). The animals treated with cortisone from day 21 following transplantation all showed prompt tumour inhibition, a greater effect being observed at the higher dose level. In animals treated with cortisone after the tumour had reached a moderate size, inhibition of further

**Fig. 5.—Degree of tumour inhibition with cortisone varied with dose. Large tumours as well as small lesions affected by this hormone.**

growth occurred and some regression was also noted. The experiment was terminated on day 50.

*Experiment III*

*Group* 1. 6 control animals (transplanted tumours untreated with hormones).

*Group* 2. Cortisone, 1·25 mg. three times weekly to 6 animals from day 18 to 49.

*Group* 3. Cortisone, 1·25 mg. three times weekly to 6 animals from day 18 to 78 following transplantation.

FIG. 6.—Initial tumour growth rate resumed when cortisone was withdrawn. Almost complete tumour inhibition achieved with a combination of Provera and cortisone.

*Group* 4. Cortisone, 1·25 mg. together with Provera 2·5 mg. three times weekly to 6 animals from day 20 to 40.

*Results.*—Tumour grafts were successful in all 30 hamsters. In the controls rapid sustained tumour growth occurred which necessitated killing the animals on day 40 (group 1 in Fig. 6). Cortisone reduced tumour growth rate which, however, resumed its original pattern when the hormone was suspended on day 49. The most striking effect was produced by the combination of cortisone and Provera which resulted in almost complete tumour inhibition (group 4 in Fig. 6). Five of the 6 animals treated by these two hormones showed no evidence of tumour growth beyond the palpable stage. In the remaining animal only very slight growth took place. The hamsters treated with cortisone and Provera all showed signs of general toxicity by day 38 and the experiment was terminated on day 40.

Inhibition of the hamster renal tumour by cortisone may be due to a specific anti-tumour effect, or to a more general inhibitory action on tumours in this species. Another possible explanation is that the doses of cortisone employed in these experiments were toxic and tumour inhibition was merely secondary to somatic disturbance in the host. Experiments IV–VII were undertaken to study these questions.

*Experiment IV*

Cortisone toxicity test.

*Group* 1. Cortisone, 2·5 mg. subcutaneously three times weekly to 5 non-tumour bearing male hamsters for 37 days.

*Group* 2. Cortisone, 1·25 mg. subcutaneously three times weekly to 5 animals similar to those in group 1 for 37 days.

*Group* 3. Liberal diet of routine food-stuff (10 g. daily) to each of 5 animals bearing transplanted renal tumours for 43 days.

*Group* 4. Restricted diet of similar food (5 g. daily) to each of 5 animals bearing transplanted renal tumours for 43 days.

*Results.*—The cortisone-treated animals appeared to remain generally well, but those receiving the higher dose (group 1) lost weight, the average at the end of the experiment being 103 g. compared with 111 g. before treatment (Fig. 7*a*). During this time hamsters of the same strain and age and kept on the same diet without treatment would be expected to increase their weight by approximately 5 per cent. The weight of the animals on the lower dose of cortisone (group 2) remained stationary (Fig. 7*a*). The carcass weight of 3 of the 5 tumour-bearing animals receiving the liberal diet (group 3) was between 10–19 per cent lower than the original body weight ; in 2 animals the weight had increased by 2 and 11 per cent respectively. The tumours of all these 5 animals grew well. The carcasses of the animals on a restricted diet (group 4) had lost between 14 and 30 per cent (average 23 per cent) of the original body weight. The growth rate and the final diameters of their tumours was approximately 20 per cent less than in the control animals receiving the liberal diet. Increasing tumour size in the presence of a rising or falling animal weight is shown in Fig. 7*b*.

It is concluded that the marked tumour inhibition observed in our experiments with cortisone treatment cannot be explained by non-specific effects of cortisone on body weight. Sparks *et al.* (1955) also tried to evaluate the anti-tumour effect of cortisone in the presence of general weight loss in C3H mice bearing transplanted mammary tumours : the administration of pituitary growth hormone (S.T.H.) with cortisone prevented body weight loss, but not tumour inhibition.

In order to try and determine whether the cortisone inhibition of the transplanted renal tumour in the hamster was due entirely to a non-specific tumour

FIG. 7(*a*).

FIG. 7*a* and *b*.—Cortisone toxicity test and effect of restricted diet on body weight and tumour size : hamsters treated with these doses of cortisone remained generally well, but at the high level there was some loss of body weight. In animals on a restricted diet well-marked tumour growth occurred in spite of loss of body weight.

effect in this species, a renal tumour of different aetiology and also two unrelated tumours arising from other tissues were studied for cortisone inhibition (experiments V, VI and VII).

*Experiment V*

The tumour in this experiment was the transplantable soft tissue sarcoma C.B. 4460 which was originally induced in hamsters treated in the Chester Beatty

Research Institute with an iron-dextran preparation (" Imferon ") and which was now in its twenty-second generation. This tumour gives a high proportion of successful takes and grows rapidly.

*Group* 1. 6 control animals (transplanted tumours untreated with hormones).

*Group* 2. Cortisone, 1·25 mg. three times weekly to 6 animals from day 7 following transplantation.

EFFECT OF DIET ON BODY WEIGHT AND TUMOUR SIZE (EXPERIMENT IV)

FIG. 7 (*b*).

*Group* 3. Cortisone, 1·25 mg. together with Provera 2·5 mg. three times weekly to 6 animals from day 7.

*Group* 4. Provera, 2·5 mg. three times weekly to 6 animals from day 7,

*Results.*—Tumour grafts were successful in all 24 animals and in the controls grew rapidly. Cortisone and Provera, alone or in combination, had no influence whatsoever on this tumour (Fig. 8) and the experiment was terminated on day 20.

Transplanted Imferon Induced Sarcoma (CB 4460) in Male Hamsters (Experiment V)

EFFECT OF PROVERA, CORTISONE AND CORTISONE + PROVERA

FIG. 8.—Administration of cortisone and cortisone together with Provera had no effect on a non-renal transplanted tumour in the hamster (sarcoma C.B. 4460).

*Experiment VI*

The transplantable hepatoma of the hamster, obtained originally from Dr. Hadley Kirkman, was used in this experiment. This tumour also gives a high proportion of successful takes and grows very rapidly.

*Group* 1. 4 control animals (transplanted tumours untreated with hormones).

*Group* 2. Cortisone, 1·25 mg. three times weekly to 4 animals from day 12 following transplantation.

Transplanted Hepatoma in Male Hamsters – (Experiment VI)

EFFECT OF PROVERA, CORTISONE AND CORTISONE + PROVERA

FIG. 9.—Administration of cortisone and cortisone together with Provera had no effect on a second non-renal transplanted tumour in the hamster (hepatoma).

*Group* 3. Cortisone, 1·25 mg. together with Provera 2·5 mg. three times weekly to 4 animals from day 12.

*Group* 4. Provera, 2·5 mg. three times weekly to 4 animals from day 12.

*Results.*—Tumour grafts were successful in all 16 animals. As in the previous experiment no difference was noted between the treated and control animals (Fig. 9) and the experiment was terminated on day 26.

*Experiment VII.* (In conjunction with Dr. F. C. Chesterman)

Spontaneous renal tumours in the hamster have not been seen in this Laboratory nor by Dr. Kirkman at Stanford University. At the time of our experiments the only other type of renal tumour reported in the hamster was that described by Stewart *et al.* (1957) which was induced by a polyoma virus isolated from

Transplanted MHP Virus Induced Kidney Sarcoma (Experiment VII : by Dr. F. C. Chesterman)

EFFECT OF CORTISONE

FIG. 10.—(*a*) Moderate doses of cortisone were without effect on the hamster transplanted polyoma renal tumour.

leukaemic mouse tissue. Negroni *et al.* (1959) isolated a virus with similar properties from the spleen of an AK mouse with spontaneous lymphocytic leukaemia (Mill Hill polyoma virus) and which, following inoculation into new born hamsters and other rodents, produced a variety of tumours. In the hamster obvious tumours were found most frequently in the kidney, heart and liver. Over 90 per cent of these animals developed renal tumours which began to appear as early as 3 days after virus inoculation (Chesterman, 1961). These tumours are rapidly growing, spindle cell sarcomas and can be serially transplanted.

At our request Dr. F. C. Chesterman of the Imperial Cancer Research Fund Laboratories at Mill Hill studied the effect of administering cortisone to hamsters

Transplanted MHP Virus Induced Kidney Sarcoma (Experiment VII : by Dr. F.C. Chesterman)

EFFECT OF CORTISONE

FIG. 10.—(*b*) Inhibition of this tumour was achieved only with doses of cortisone which were toxic, causing marked general weight loss and death of the host.

bearing the transplanted Mill Hill polyoma virus renal tumour which was in its thirty-fifth transfer generation. Twenty male golden hamsters from a colony bred in the Imperial Cancer Research Fund Laboratory were treated with 5 mg. of cortisone (Roussel) per 100 g. body weight subcutaneously on day 5, 7, 9 and 11 after tumour inoculation. With this dose no effect on tumour growth was observed (Fig. 10*a*). Because of the extremely rapid growth of the tumour the experiment had to be terminated on day 13. During the period of observation the animals showed no toxic effects. It was only in further experiments with more prolonged cortisone treatment (5 mg. per 100 g. body weight on day 6, 8, 10, 12, 15 and 19) leading to marked general toxic effects associated with considerable weight loss that tumour inhibition was noted (Fig. 10*b*). These animals had to be killed at intervals soon after day 14, either because of large tumour size or marked cortisone toxicity ; the experiment was finally terminated on day 26. It would appear that, during the short period of observation, cortisone, in doses tolerated by the hamster, had no effect on the transplanted polyoma renal tumour.

### *Summary of Renal Tumour Histology in Hormone-Treated Hamsters*

Transplanted adenocarcinomas in control hamsters and in those treated with testosterone or Provera all showed signs of active proliferation with limited central necrosis comparable to that found in untreated controls. On the other hand, the tumours from animals treated with cortisone, alone or in combination

---

### EXPLANATION OF PLATES

FIG. 11.—(*a*) Multiple bilateral primary adenomatous tumours in the renal cortex of a stilboestrol-treated male hamster.

(*b* and *c*) Unusually large multiple adenomas in the renal cortex of a male patient aged 51 in whom nephrectomy was carried out for chronic pyelonephritis.

FIG. 12.—(*a*) Mr. G. D. aged 64 (042760) multiple bilateral pulmonary metastases 2 months following nephrectomy for adenocarcinoma of renal parenchyma. Solitary lung deposit was present before operation.

(*b*) Radiological disappearance of all but two deposits in right lung, 8 weeks after commencing treatment with Provera, 300 mg. daily by mouth. These deposits remained stationary for 10 months and then slowly increased in size in spite of further treatment with Provera, prednisone and finally testosterone.

FIG. 13.—(*a*) Mr. D. W. aged 58 : (040856), skeletal and intrathoracic metastases at time of nephrectomy for adenocarcinoma of the renal parenchyma with invasion of the renal vein. Pain and cachexia present 6 weeks post-operatively. Deterioration in general condition together with increase in size of metastases in spite of treatment with oral Provera 300-500 mg. daily for 2 months.

(*b*) Striking improvement in general condition occurred within 2 months of changing from Provera by mouth to testosterone proprionate by injection : skeletal and intrathoracic metastases became smaller after 7 months treatment. Patient shown here after 18 months testosterone therapy : 42 pounds gain in weight. Remained well on maintenance dose of methyl testosterone 50 mg. daily sublingually with no evidence of active malignant disease for 3 years after commencing hormone treatment. Recently, new metastases have appeared.

(*c*) Osteolytic deposit in cranial vault increasing in size during Provera treatment.

(*d*) Healing of skull defect 18 months after changing from Provera to testosterone. Histological proof of metastases obtained from tibial deposit.

FIG. 14.—Mr. W. D. aged 59, (No. 049868), a recent case kindly referred by Sir Eric Riches : Pulmonary metastases and a large fixed renal tumour which was initially treated by irradiation. At laparotomy 4 weeks later renal mass still completely fixed and metastases seen in liver and on diaphragm. Biopsy of secondary diaphragmatic nodule only performed—renal adenocarcinoma. Pulmonary metastases increased in size and number and patient's general condition deteriorated (*a*).

Five weeks after commencing provera, 300 mg. daily by mouth, the patient was improved. Chest X-ray showed disappearance of pulmonary metastases (*b*).

11a

11b

11c

12a

12b

14a

14b

13a

13c

13b

13d

Bloom, Dukes and Mitchley.

with Provera, all showed extensive areas of necrosis and haemorrhage. In the areas adjacent to necrosis reduced mitotic activity and varying degrees of tumour cell degeneration, accompanied by an inflammatory reaction, were seen. Small residual areas of viable-looking tissue remained, however, at the tumour periphery in all these animals. Metastases were not observed in any of the treated or control animals.

No histological difference was found between the tumours of treated and untreated hamsters bearing either the transplanted C.B. 4460 sarcoma or the transplanted hepatoma.

### DISCUSSION

In the preceding experiments Provera and also testosterone had no effect on the growth of the transplanted stilboestrol-induced renal tumour in the male hamster. Cortisone, on the other hand, in moderate doses induced extensive areas of tumour necrosis accompanied by a marked reduction in growth rate. In general, the inhibitory effect of hormones upon experimental animal tumours is cessation of further development, or merely reduction in growth rate. Well marked regression of an established tumour is a rare event, and this has been our experience with the hamster renal tumour. Some degree of regression, however, was observed with prolonged cortisone treatment (Fig. 4 and 5).

Whereas progesterone inhibited the induction of the primary renal tumour by stilboestrol and Professor Horning believed that Provera may inhibit the transplanted oestrogen-*dependent* tumour, the latter preparation, in doses of 2·5 mg. two to three times weekly, in our experiments failed to influence the transplanted oestrogen-*independent* growth.

The physiological and anti-tumour action of steroid substances are not necessarily related, and small differences in molecular structure of closely allied compounds may result in marked differences in the effects they produce. Thus, although the physiological activity of Provera is claimed to be many times greater than that of progesterone in its action, for example, on endometrium, this may be quite unrelated to its potency as an anti-tumour agent. We have not found any reports in the literature concerning the effect of Provera on animal and human tumours, but at the time of our hamster experiments we were also testing this compound in patients with endometrial carcinoma, a tumour known to respond in a proportion of cases to progesterone and to certain related synthetic preparations (Kelley and Baker, 1961 ; Stoll, 1961). So far we have seen an objective response in one of 4 patients with advanced endometrial cancer treated with 300 mg. of Provera orally, daily (Bloom and Speed, 1963, unpublished observations). In this patient there was complete disappearance of pulmonary metastases within eight weeks of commencing treatment, and this has been maintained for eighteen months.

In collaboration with Dr. F. J. C. Roe we are now investigating the effect of Provera in doses larger than those employed in the animal experiments reported here, and also of other progestational agents on the oestrogen-*independent* as well as the oestrogen-*dependent* transplanted renal tumour. Provera in daily doses of 0·625–2·5 mg. reduces growth rate and produces necrosis of the transplanted tumour which is still dependent on oestrogen administration for its sustained growth. Inhibition of the oestrogen-*independent* tumour was ultimately achieved with much larger doses of Provera (20 mg. and 40 mg. daily) than were used in the present experiments.

The inhibitory effect of cortisone on the hamster renal tumour is not due to a general toxic effect producing loss of body weight in the host. Furthermore, the action of this hormone has some degree of specificity in that no such inhibition was noted with the transplanted polyoma renal tumour in the hamster, nor on two unrelated non-renal tumours in the same species. On the other hand, Crabb and Kelsall (1951) found that cortisone acetate in doses of 0·15 mg./100 g. body weight administered to male hamsters during a period preceding and following grafting caused growth retardation of quite a different tumour—a transplanted lung sarcoma.

A rapidly growing transplanted renal adenocarcinoma which arose spontaneously in a male hamster in 1957 has been carried on in serial transplants by Fortner *et al.* (1961), and the effect on this tumour of various chemotherapeutic agents and hormones, including cortisone, has been studied recently by Schabel *et al.* (1961). No anti-tumour effect was observed with this hormone in doses up to 35 mg. per kilogramme body weight per day for 7 days. The influence of cortisone on 17 other different transplantable tumours in the hamster was also studied. Marked inhibition was noted in only two examples, a melanoma and a plasmacytoma. It would be of interest to study the influence of cortisone and gonadal hormones on induced adenomatous renal tumours in other species such as the one associated with lead acetate administration in the rat (Boyland *et al.*, 1962).

As with hormone dependent tumours in general, resistance may ultimately develop in a tumour previously sensitive to cortisone (Lampkin and Potter, 1958). We found no loss of inhibitory action of this hormone on the transplanted renal tumour carried over five serial transplantations.

A combination of cortisone and Provera produced a greater suppression of transplanted renal tumour growth than was achieved with cortisone alone (Fig. 6). This observation, which was confirmed in a second series of experiments, is of particular interest in view of recent reports that a substance, chemically related to progesterone, delta-l-testololactone (Fried *et al.*, 1953), can potentiate the physiological action of certain hormones, such as testosterone, in castrated immature male mice (Lerner *et al.*, 1960) and also the anti-tumour effect of cortisone in mouse mammary tumours (Woolley, 1960). Delta-1-testololactone itself is physiologically inert, but possesses an anti-tumour effect on human breast cancer (Segaloff *et al.*, 1960) and is derived from progesterone and other steroids by a process of bio-synthesis. Experiments are being undertaken in this Laboratory to investigate the effect of this substance, alone and in combination with other hormone preparations, on the transplanted hamster renal tumour.

After six years of repeated transfer the hamster renal tumour is independent of stilboestrol-pre-treatment of the host. Further experiments, reported in our following paper (Bloom, Baker, Dukes and Mitchley, 1962), have shown that bilateral adrenalectomy or castration inhibits this tumour. The effect is greater with castration and can be nullified by the administration of either oestradiol monobenzoate or testosterone proprionate. It is likely, therefore, that the transplanted renal tumour is still oestrogen-dependent, being maintained by endogenous oestrogen derived from the testis (Goldzicher and Roberts, 1952 ; Huggins and Moulder, 1945) and from the adrenal. The action of testosterone can perhaps be explained by its conversion in the body to oestrogen (West *et al.*, 1956; Braun-Cantilo *et al.*, 1962). On the other hand, attempts to substitute androgen for oestrogen in the induction of renal tumours in male hamsters by implanting 30 mg. testosterone proprionate subcutaneously were not followed by the appearance of renal tumours after 900 days observation (Kirkman, 1958).

Ghaleb (1961) found that tritium-labelled diethyl-stilboestrol was taken up by renal tubular epithelium in the hamster and that the concentration of protein-bound stilboestrol was significantly higher in males than in females. The action of certain carcinogenic compounds may be related to their combination with cellular protein in the target tissue. The tumour inhibitory action of cortisone may, therefore, depend upon competition between this hormone and endogenous oestrogen for combination with protein in tumour cells. A greater affinity for cortisone may exclude oestrogen from these cells, leading either to their death or to their increased differentiation. Our colleague, Dr. E. J. Ambrose (1960) has found that the addition of cortisone in a concentration of $2 \times 10^{-4}$ g./ml. to the media in which hamster renal tumour cells are being cultured tends to restore to these cells the structural features and the adhesive properties of the cellular membrane found in normal renal epithelium. The cortisone $LD_{50}$ for this tumour was found to be three to four times lower than the value for normal hamster renal epithelium.

In spite of the great difficulties generally encountered in trying to draw a parallel between the behaviour of animal and human tumours, we are tempted at this stage to speculate whether the induction and progress of the hamster renal tumour and adenomatous tumour of the kidney in man have a comparable basis. The origin of both tumours is closely related to renal tubules, and their macroscopic and histological appearances have a number of features in common.

One or more small adenomas are a common finding in the human kidney and occasionally these progress to form multiple large tumours, the appearance of which closely resembles that found in the stilboestrol-treated hamster (Fig. 11*a*, *b*, *c*). We have not seen adenocarcinoma nor multiple prominent adenomas of the kidney in men following prolonged oestrogen administration for carcinoma of the prostate, but proliferative cellular changes in the renal glomerular tufts of such cases, similar to those seen in stilboestrol-treated guinea-pigs, have been described by Trevan (1956). To produce renal tumours in man the duration of oestrogen treatment would presumably have to extend over many years, since in the hamster such tumours do not appear until after 7 to 9 months' treatment which represents approximately one-third of the animal's total life-span.

We have referred to the influence of various hormones, especially those of gonadal origin on the normal kidney; to the role of the kidney itself as an endocrine organ producing "angiotensin" and "erythropoietin", and also to reasons for suspecting that renal adenocarcinoma in man, as in the hamster, may be influenced by hormonal factors. Since hormones possess a marked degree of tissue specificity and since their principal actions are fundamentally alike in all species, knowledge gained from employing these substances in animal experiments is worth exploring in man.

Since May 1959, 17 patients with metastases from adenocarcinoma of the kidney have been treated with hormones at the Royal Marsden Hospital and this work is to be the subject of a separate clinical report. In brief, there have been three cases with pulmonary metastases which have shown an objective response to Provera and a further patient with skeletal deposits who responded quite dramatically to testosterone, having first failed to improve with Provera (Fig. 13). The response following testosterone in this case was maintained for over three years. In other patients metastases have appeared to remain stationary for a time or to progress more slowly since commencing or changing hormone therapy.

Caution in interpreting these observations is, of course, necessary since the natural slow progress or actual regression of metastases are well-known features of renal cancer in man (Miller *et al.*, 1962 ; Gordon and Bateson, 1962). Partial or complete spontaneous regression in human cancer, however, is an exceedingly rare event. Everson and Cole (1956) were able to find only 47 authentic examples of this phenomenon in the literature and by personal enquiry : only 2 of these cases had hypernephromas. In cancer of the breast, a tumour of established hormone dependency, there were no examples of spontaneous regression in 250 untreated cases studied by Bloom *et al.* (1962) and fluctuation in tumour growth rate was noted in only one patient. Regression on disappearance of metastases in the four cases of renal tumour referred to occurred within such a short time of administering or changing the hormone preparation that it seemed likely that the effect was related to treatment and not to a spontaneous event. Further support for a therapeutic effect is found in the case showing regression of bone metastases under testosterone therapy since, as far as we know, spontaneous regression of skeletal deposits in this disease has not been reported. All examples in the literature of spontaneous regression of renal adenocarcinoma have been in relation to pulmonary deposits. Histological proof of metastases was obtained in 3 of our 4 patients showing regression under hormone treatment. The fourth patient died elsewhere and an autopsy was not performed.

It should be noted that whereas Provera and also testosterone each failed to influence tumour growth in the present experiments with the transplanted hamster renal tumour, the administration of these hormones to patients with metastatic renal adenocarcinoma was associated with objective signs of tumour regression. In subsequent hamster experiments, however, Provera was found to inhibit the stilboestrol-*dependent* strain of transplantable renal tumour and, using much greater doses of this hormone, restraint of the " independent " growth itself was finally achieved. So far, only those patients who have failed to respond to Provera or to testosterone or who have escaped from such hormonal control, have been treated with a cortico-steroid. Prednisone was used in a small number of cases but so far no obvious tumour regression has been observed with this preparation, although in the hamster cortisone produced marked regression of the transplantable renal tumour. On the other hand, according to Kirkman (1959) cortisone increased the incidence of primary kidney tumours in stilboestrol-treated hamsters, and also favoured the development of metastases in stilboestrol-treated hosts bearing transplanted tumours.

Human cancer that responds to changes in hormonal environment generally arises in tissues which are normally under endocrine influence such as the breast, thyroid, prostate and body of uterus. It would, therefore, be of great interest if the development and progress of tumours of the human kidney eventually proved to be related to the endocrine system. It is of additional interest that the hormones concerned with renal tumour induction and inhibition in the hamster, and associated with regression of metastatic cancer of the kidney in man, are of sex origin.

The bridge with which we have sought to span the experimental results in hamsters, and the clinical observations in patients showing regression or reduced growth rate of renal tumour metastases whilst receiving hormone therapy is slender and more work needs to be done before one can speak of hormone-dependency in human renal cancer. Further information concerning this question may be obtained, for example, from the hormone excretion pattern in the urine of patients with adenocarcinoma of the kidney before and after removal of the primary tumour, when metastases appear and during hormone therapy. An investigation of this type has been undertaken at the Royal Marsden Hospital and attention is being given to oestrogen, 17-ketosteroid, 17-hydroxyketosteroid and gonadotrophin excretion. More important, however, is the opportunity to observe a larger number of cases under treatment and in the meantime this paper is presented in the hope that other workers will now seek to test hormone-responsiveness in those patients with advanced carcinoma of the kidney for whom no other treatment is practical.

### SUMMARY

The development, natural history and pathological features of the oestrogen-induced renal adenocarcinoma of the golden hamster reported originally by Dr. Hadley Kirkman and by the late Professor E. Horning, have been reviewed. The factors known to influence tumour induction and subsequent growth have been described.

A series of new experiments are reported in which the effect of three hormone preparations on tumour growth rate have been observed using the *transplanted* renal tumour in young male hamsters. This tumour, in its 21st–35th generations, progresses rapidly and is independent of exogenous oestrogen administration.

A synthetic oral progestational agent, 6-alpha-methyl-17-alpha-hydroxy-progesterone acetate (Provera), in doses of 2·5 mg. subcutaneously three times weekly, had no effect on tumour growth compared with untreated controls. Testosterone proprionate in similar dosage also had no effect. On the other hand, a marked inhibitory effect was seen in the tumours of those animals treated with cortisone in doses of 1·25–2·5 mg. subcutaneously three times weekly, the reduction in tumour growth rate being greater with the larger dose. This effect was not dependent upon a general toxic manifestation in the last and, indeed, appeared to have some specificity for the kidney tumour in that no changes were observed with cortisone treatment in a transplanted polyoma kidney tumour, nor in two unrelated non-renal tumours.

The most striking effect on the hamster renal tumour was achieved by a combination of cortisone and Provera. These hormones together resulted in practically complete inhibition of tumour growth, whereas Provera alone in the same dosage, failed to bring about a tumour response. This observation, which was confirmed in more recent experiments, is of interest because of the possible hormone-potentiating effect of delta-1-testololactone, a substance related to progesterone, but physiologically inert.

Hormones possess a marked degree of tissue specificity and their principal actions are fundamentally alike in all species. Chiefly because of this fact and the existence of certain gross and microscopic similarities between the adenomatous renal tumours of the hamster and adenoma and adenocarcinoma of the human kidney, we have been tempted to consider that these animal experiments may shed light on the aetiology of renal carcinoma in man and point to a possible new treatment for advanced cases.

Reference has been made to a clinical experiment being undertaken at the Royal Marsden Hospital in which the effect of hormone administration to patients with disseminated renal adenocarcinoma (hypernephroma) is being studied. In some cases objective signs of tumour inhibition or of actual regression have been noted with Provera or testosterone and, at the present time, these observations are considered more likely to be related to the endocrine treatment than to a spontaneous event.

It is of interest that hormone dependent tumours in the hamster and possibly in man may arise in the kidney, an organ which is not a recognised member of the endocrine system nor a secondary sex organ. It is of additional interest that the hormones concerned with primary renal tumour induction and inhibition in the hamster, and associated with regression of metastatic carcinoma of the kidney in man, are of sex origin. On the other hand, a renotropic action of certain gonadal hormones has been known for many years following the original observations that castration and androgen administration influence kidney size in mice and rats.

The kidney is a target organ for hormones other than growth hormone, pitressin and corticosteroids and, by virtue of "angiotensin" and "erythropoietin" production, appears to have a role to play as an endocrine gland itself. It is now suggested that a relationship may exist between hormonal factors and the development and progress of the renal parenchymal adenoma and adenocarcinoma in man.

We are grateful to Professor Alexander Haddow for his interest in this work and to Dr. Francis J. C. Roe for helpful advice in the preparation of the manuscript.

The investigation has been supported by grants to the Chester Beatty Research Institute from the Medical Research Council, the British Empire Cancer Campaign, the Anna Fuller Fund, and the National Cancer Institute of the National Institutes of Health, U.S. Public Health Service.

We should like to thank Dr. N. F. C. Gowing for Fig. 11*b* and 11*c* and the departments of Medical Art and Photography of the Royal Marsden Hospital for the illustrations.

We are indebted to Dr. R. G. Jacomb of Upjohn Ltd for the supplies of Provera.

### REFERENCES

AGNEW, L. R. C. AND GARDNER, W. U.—(1952) *Cancer Res.*, **12**, 757.
AMBROSE, E. J.—(1960) *Rep. Brit. Emp. Cancer Campgn.*, **38**, 96.
BABCOCK, J. C., GUTSELL, E. S., HERR, M. E., HOGG, J. A., STUCKI, J. C., BARNES, L. E. AND DULIN, W. E.—(1958) *J. Amer. chem. Soc.*, **80**, 2904.
BASERGA, R. AND SHUBIK, P.—(1954) *Cancer Res.*, **14**, 12.
BIELSCHOWSKY, F. AND HORNING, E. S.—(1958) *Brit. med. Bull.*, **14**, 106.
BLOOM, H. J. G.—(1960) *Rep. Brit. Emp. Cancer Campgn*, **38**, 95.
*Idem*, BAKER, W. H., DUKES, C. E. AND MITCHLEY, B. C. V.—(1963) *Brit. J. Cancer*, **17**, 646.
*Idem*, RICHARDSON, W. W. AND HARRIES, E. J.—(1962) *Brit. med. J.*, ii, 213.
BOYLAND, E., DUKES, C. E., GROVER, P. L. AND MITCHLEY, B. C. V.—(1962) *Brit. J. Cancer*, **14**, 283.

BRAUN-CANTILO, J. A., LA ROCHE, G., NOVITSKY, M. AND LAWRENCE, J. H.—(1962) *Acta Isotopica*, **1**, 351.
BURCHENAL, J. H., STOCK, C. C. AND RHOADS, C. P.—(1950) *Cancer Res.*, **10**, 209.
CHESTERMAN, F. C.—(1961) *Med. Press*, **245**, 350.
*Idem*, FRANKS, L. M., KNUDSEN, E. T. AND WILLIAMS ,P. C.—(1956) *Lancet*, ii, 1192.
CONLEY, C. L., KOWAL, J. AND D'ANTONIO, J.—(1957) *Johns Hopk. Hosp. Bull.*, **101**, 63.
COOK, J. W. AND DODDS, E. C.—(1933) *Nature, Lond.*, **31**, 205.
CRABB, E. D. AND KELSALL, M. A.—(1951) *Cancer Res.*, **11**, 243.
CRABTREE, C. E.—(1941) *Endocrinology*, **29**, 197.
CRISTOL, D. S., MCDONALD, J. R. AND EMMETT, J. L.—(1946) *J. Urol.*, **55**, 18.
DEWEERD, J. H. AND HAGEDORN, A. B.—(1959) *Ibid.*, **82**, 29.
DMOCHOWSKI, L. AND HORNING, E. S.—(1940) *J. Path. Bact.*, **59**, 307.
EVERSON, T. C. AND COLE, W. H.—(1956) *Ann. Surg.*, **144**, 366.
FISHMAN, W. H.—(1951) *Ann. N.Y. Acad. Sci.*, **54**, 548.
*Idem*, ARTENSTEIN, M. AND GREEN, S.—(1955) *Endocrinology*, **57**, 646.
*Idem* AND FARMELANT, M. H.—(1953) *Ibid.*, **52**, 536.
FORTNER, J. G., MAHY, A. G. AND COTRAN, R. S.—(1961) *Cancer Res.*, **21** (Part 2), 99.
FRANKS, L. M.—(1954) *J. Path. Bact.*, **68**, 603.—(1956) *Lancet*, ii, 1037.
FRIED, J., THOMA, R. W. AND KLINGSBERG, A.—(1953) *J. Amer. chem. Soc.*, **75**, 5764.
GARDNER, W. U.—(1948) *Cancer Res.*, **8**, 397.
GARDNER, W. U.—(1953) in ' Advances in Cancer Research ', edited by Greenstein, J. P. and Haddow, A., **1**, 173, N. York (Acad. Press).
*Idem*, DOUGHERTY, T. F. AND WILLIAMS, W. L.—(1944) *Cancer Res.*, **4**, 73.
*Idem*, KIRSCHBAUM, A. AND STRONG, L. C.—(1940) *Arch. Path.*, **29**, 1.
GHALEB, H. A.—(1961) Ph.D. Thesis, University of London.
GOLDENBERG, I. S. AND HAYES, M. A.—(1959) *Cancer*, **12**, 738.
GOLDZICHER, J. W. AND ROBERTS, I. S.—(1952) *J. clin. Endocrin.*, **12**, 143.
GORDON, D., HORWITT, B. N., SEGALOFF, A., MURISON, P. J. AND SCHLOSSER, J. V.—(1952) *Cancer*, **5**, 275.
GORDON, F. M. AND BATESON, E. M.—(1962) *Brit. J. Radiol.*, **35**, 425.
GOTTSCHALK, R. G. AND GROLLMAN, A.—(1952) *Cancer Res.*, **12**, 651.
GRIFFITHS, I. H. AND THACKRAY, A. C.—(1949) *Brit. J. Urol.*, **21**, 128.
GURNEY, C. W., JACOBSON, L. O. AND GOLDWASSER, E.—(1960) in ' Clinical Endocrinology ', edited by Astwood, E. B., **1**, 592, New York (Grune and Stratton).
HADDOW, A.—(1947) *Brit. med. Bull.*, **4**, 331.
HEILMAN, F. R. AND KENDALL, E. C.—(1944) *Endocrinology*, **34**, 416.
HEIMAN, J.—(1940*a*) *Amer J. Cancer*, **39**, 172.—(1940*b*) *Ibid.*, **39**, 178.—(1940*c*) *Ibid.*, **40**, 343.—(1943) *Cancer Res.*, **3**, 65.
HEWLETT, J. S., HOFFMAN, G. C., SENHAUSER, D. A. AND BATTLE, J. D.—(1960) *New Engl. J. Med.*, **262**, 1058.
HIGGINS, G. M., WOODS, K. A. AND BENNETT, W. A.—(1950) *Cancer Res.*, **10**, 203.
HORNING, E. S.—(1952) *Rep. Brit. Emp. Cancer Campgn.*, **30**, 60.—(1954) *Brit. J. Cancer*, **8**, 627.—(1955) *Rep. Brit. Emp. Cancer Campgn.*, **33**, 62.—(1956*a*) *Brit.*, *J. Cancer*, **10**, 678.—(1956*b*) *Z. Krebsforsch.*, **61**, 1.—(1957) *Lect. Sci. Basis Med.*, **5**, 421.
*Idem* AND WHITTICK, J. W.—(1954) *Brit. J. Cancer*, **8**, 451.
HUGGINS, C. AND MOULDER, P. V.—(1945) *Cancer Res.*, **5**, 510.
*Idem*, BERGENSTAL, D. M. AND CLEVELAND, A. S.—(1953) *Recent Progr. Hormone Res.*, **8**, 273.
*Idem*, TORRALBA, Y. AND MAINZER, K.—(1956) *J. exp. Med.*, **104**, 525.
INGLE, D. J. AND NEZAMIS, J. E.—(1951) *Endocrinology*, **48**, 484.
ISING, U.—(1956) *Acta path. microbiol. scand.*, **39**, 168.
JACOBSON, L. O., GOLDWASSER, E., FRIED, W. AND PLZAK, L.—(1957) *Nature, Lond.*, **179**, 633.

JONSSON, U., COLSKY, J., LESSNER, H. E., ROATH, O. S., ALPER, R. G. AND JONES, R.—(1959) *Cancer*, **12**, 509.
KELLY, R. M. AND BAKER, W. H.—(1961) *New Engl. J. Med.*, **264**, 216.
KIRKMAN, H.—(1951) *Anat. Rec.*, **109**, 51.—(1957) *Cancer*, **10**, 757.—(1958) *Rep. Brit. Emp. Cancer Campgn*, **36**, 44.—(1959) *Nat. Cancer Inst.*, Monograph, No. 1.
*Idem* AND BACON, R. L.—(1949) *Anat. Rec.*, **103**, 475.—(1952*a*) *J. nat. Cancer Inst.* **13**, 745.– (1952*b*) *Ibid.*, **13**, 757.
*Idem* AND HORNING, E.—(1957) *Rep. Brit. Emp. Cancer Campgn*, **35**, 66.
*Idem* AND ROBBINS, M.—(1959) *Nat. Cancer Inst.*, Monograph, No. 1, p. 93.
*Idem* AND WURSTER, D. H.—(1957) *Proc. Amer. Ass. Cancer Res.*, **2**, 221.
KOCHAKIAN, C. D., BARTLETT, M. N. AND GORGORA, J.—(1948) *Amer. J. Physiol.*, **153**, 210.
KORENCHEVSKY, V. M. AND DENNISON, M.—(1934) *J. Path. Bact.*, **38**, 231.—(1935) *Proc. R. Soc. Med.*, **28**, 1265.
*Idem*, DENNISON, M. AND KOHN-SPEYER, A.—(1933*a*) *Biochem. J.*, **27**, 557.—(1933*b*) *Ibid.*, **27**, 1506.
*Idem* AND ROSS, M. A.—(1940) *Brit. med. J.*, i, 645.
LACASSAGNE, A.—(1938) *Bull. Ass. franç. Cancer*, **27**, 96.—(1957) Proc. 2nd Canad. Cancer Conf., 1955, N. York (Acad. Press) p. 267.
LAMPKIN, J. MC. AND POTTER, M.—(1958) *J. nat. Cancer Inst.*, **20**, 1091.
LATTIMER, J. K.—(1942) *J. Urol.*, **48**, 778.
LEARY, T.—(1950) *Arch. Path.*, **40**, 151.
LEMON, H. M.—(1957) *Ann. intern. Med.*, **46**, 457.—(1959) *Cancer*, **12**, 93.
*Idem* AND SMAKULA, E.—(1955) *Cancer Res.*, **15**, 273.
LERNER, L. J., BIANCHI, A. AND BORMAN, A.—(1960) *Cancer*, **13**, 1201.
LIPSCHUTZ, A., MURILLO, R. AND VARGAS, L.—(1939) *Lancet*, ii, 420.
*Idem* AND VARGAS, L.—(1941) *Endocrinology*, **28**, 669.
LUDDEN, J. B., KRUEGER, E. AND WRIGHT, I. S.—(1941) *Ibid.*, **28**, 619.
MCALPINE, R. N., BLAIR, S. M., GILLIES, D. R., LYONS, W. R. AND LI, C. H.—(1958) *Cancer*, **11**, 731.
MACKAY, E. M.—(1940) *Proc. Soc. exp., Biol., N.Y.*, **45**, 216.
MCQUEEN-WILLIAMS, M. AND THOMPSON, K. W.—(1940) *Yale J. Biol. Med.*, **12**, 531.
MARTINEZ, C., VISSCHER, M. B., KING, J. T. AND BITTNER, J. J.—(1952) *Proc. Soc. exp. Biol., N.Y.*, **80**, 81.
MATTHEWS, V. S., KIRKMAN, H. AND BACON, R. L.—(1947) *Ibid.*, **66**, 195.
MILLER, E. W., ORR, J. W. AND PYBUS, F. C.—(1943) *J. Path. Bact.*, **55**, 137.
MILLER, H. C., WOODRUFF, M. W. AND GAMBACORTA, J. P.—(1962) *Ann. Surg.*, **156**, 852.
MURPHY, J. B. AND STURM, E.—(1944) *Science*, **99**, 303.
NAETS, J. P.—(1958) *Nature, Lond.*, **181**, 1134.
NEGRONI, G., DOURMASHKIN, R. AND CHESTERMAN, F. C.—(1959) *Brit. med. J.*, ii, 1359.
NEWCOMBE, W. D.—(1937) *Proc. R. Soc. Med.*, **30**, 113.
NOBLE, R. L.—(1957) *Pharmacol. Rev.*, **9**, 367.
*Idem* AND COLLIP, J. B.—(1941) *Canad. med. Ass. J.*, **44**, 1.
PAGE, I. H. AND BUMPUS, M.—(1960) in ' Clinical Endocrinology ', edited by Astwood, E. B. New York (Grune and Stratton), Vol. 1, p. 591.
PEARSON, O. H., LI, M. C., MACLEAN, J. P., LIPSETT, M. B. AND WEST, C. D.—(1955) *Ann. N.Y. Acad. Sci.*, **61**, 393.
Pfeiffer, C. A., EMMEL, V. M. AND GARDNER, W. U.—(1940) *Yale J. Biol. Med.*, **12**, 493.
PLZAK, L. F.—(1960) *Surg. Forum*, **10**, 121.
PYBUS, F. C. AND MILLER, E. W.—(1938) *Nature, Lond.*, **142**, 872.
RICHARDSON, F. L.—(1957) *J. nat. Cancer. Inst.*, **18**, 813.
SCHABEL, F. M., SKIPPER, H. E., FORTNER, J. G., THOMSON, J. R., LASTER, W. R., MOORE, J. H., KELLY, C. A. AND FARNELL, D. C.—(1961) *Cancer Res.*, **21**, Part 2, 235.

SEGALOFF, A., WEETH, J. B., MEYER, K. K., RONGONE, E. L. AND CUNNINGHAM, M. E. G.—(1960) *Cancer*, **15**, 633.
SELYE, H.—(1939) *J. Urol.*, **42**, 637.—(1940) *Canad. med. Ass. J.*, **42**, 113.—(1941) *J. Urol.*, **46**, 110.—(1950) 'The Physiology and Pathology of Exposure to Stress', Montreal (Acta Inc.) p. 603.
*Idem* AND FRIEDMAN, S. M.—(1941) *Endocrinology*, **29**, 80.
*Idem* AND HOLLETT, C.—(1945) *J. Urol.*, **53**, 498.
*Idem* AND STEVENSON, J.—(1940) *Canad. med. Ass. J.*, **42**, 188.
SHIMKIN, M. B., SHIMKIN, P. M. AND ANDERVONT, H. B.—(1963) *J. nat. Cancer Inst.*, **30**, 135.
SPARKS, L. L., DAANE, T. A., HAYASHIDE, T., COLE, R. D., LYONS, W. R. AND LI, C. H.—(1955) *Cancer*, **8**, 271.
STEWART, S. E., EDDY, B. E., GOCHENOUR, A. M., BORGHESE, N. G. AND GRUBBS, G. E.—(1957) *Virology*, **3**, 330.
STOCK, C. C.—(1952) In 'Steroid Hormones and Tumour Growth', edited by Wolstenholme, G. E. W. AND CAMERON, M. P., Ciba Foundation Colloquia on Endocrinology, Philadelphia (Blackiston and Co.) Vol. I, p. 135.
*Idem* AND SUGUIRA, K.—(1958) *Ann. N.Y. Acad. Sci.*, **76**, 720.
STOLL, B. A.—(1961) *Cancer Chemother. Rep.*, **14**, 83.
SUGUIRA, K., STOCK, C. C., DOBRINER, K. AND RHODES, C. P.—(1950) *Cancer Res.*, **10**, 244.
TAYLOR, S. G., AYER, J. P. AND MORRIS, R. S.—(1950) *J. Amer. med. Ass.*, **144**, 1058.
TREVAN, D. T.—(1956) *Lancet*, ii, 22.
TRINKLE, A. J.—(1936) *Amer. J. Cancer*, **27**, 676.
TRUNNELL, J. B., DUFFY, B. J., MARSHALL, V., WHITMORE, W. F. AND WOODARD, H. Q.—(1951) *J. clin. Endocrin.* **11**, 663.
VAN DYKE, D. C., CONTOPOULOS, A. N., WILLIAMS, B. S., SIMPSON, M. E., LAWRENCE, J. H. AND EVANS, H. M.—(1954) *Acta haemat.*, **11**, 203.
VASQUEZ-LOPEZ, E.—(1944) *J. Path. Bact.*, **56**, 1.
VOLK, H., ESCHER, G. C., HUSEBY, R. A., TYLER, F. H. AND CHEDA, J.—(1960) *Cancer*, **13**, 757.
WEST, C. D., DAMAST, B. L., SARRO, S. D. AND PEARSON, O. H.—(1956) *J. biol. Chem.*, **218**, 409.
WILLIS, R. A.—(1948) 'Pathology of Tumours', London (Butterworth and Co.), p. 453.
WINTERNITZ, M. C. AND WATERS, L. L.—(1940) *Yale J. Biol. Med.*, **12**, 705.
WOOLLEY, G. W.—(1960) In discussion following paper by Rosoff, C.B. in 'Biological Activities of Steroids in Relation to Cancer', edited by Pincus, G. and Vollmer, E. B. New York (Acad. Press), p. 382.